# Slender lobes is a spindle matrix protein regulated by spindle envelope kinase Greatwall in oocytes

**DOI:** 10.1083/jcb.202606020

**Published:** 2026-10-26

**Authors:** Rayane Kaade, Emma Peat, Charlotte Repton, Julia Niziol, Carmela Silva Mason, Li Ern Yap, Gera Pavlova, Hiroyuki Ohkura

**Affiliations:** 1 School of Biological Sciences, University of Edinburgh, Edinburgh, UK

## Abstract

Chromosome segregation is mediated by microtubule-based machinery, the spindle. Microtubule-independent structures, often called the spindle matrix or the spindle envelope, have been reported to embed or surround the spindle, respectively. The concepts have been around for several decades, but they still remain enigmatic. Here, we show the spindle matrix and the spindle envelope form one functional unit crucial for spindle structure and function in oocytes. We found that in *Drosophila* oocytes, Slender lobes (Sle) protein is an integral component of the spindle matrix, which has a composition different from that in mitotic cells. We further found that Greatwall (Gwl) kinase localizes to the spindle envelope tightly surrounding the Sle spindle matrix in oocytes. Gwl promotes assembly of the Sle spindle matrix, which in turn determines the location of the Gwl spindle envelope. *sle* silencing in oocytes resulted in spindle abnormalities. Cosilencing experiments revealed that Sle buffers the impacts of compromised kinetochore–microtubule interaction on centromere bi-orientation in meiotic metaphase I.

## Teaser

Kaade et al. identify Sle as an integral component of the spindle matrix, and the Gwl kinase as a spindle envelope protein in *Drosophila* oocytes. They work together to ensure proper structure and function of the meiotic spindle in oocytes.

## Introduction

In oocytes, the spindle is formed without centrosomes, the main microtubule-organizing centers in mitosis, and also within a large cytoplasmic volume (Dumont and Desai, 2012; Ohkura, 2015; Kyogoku and Kitajima, 2023). This lack of spatial constraints makes spindle formation challenging in oocytes. The spindle consists of microtubules and their associated proteins. However, some evidence suggests that the spindle is embedded in a microtubule-independent structure or substance, often called the spindle matrix (Pickett-Heaps et al., 1997; Tsai et al., 2006; Johansen and Johansen, 2009; Ma et al., 2009). The spindle matrix potentially provides a local environment that promotes the spindle assembly and stability, but its existence, composition, regulation, and function are still elusive.

The idea of a spindle matrix was first proposed more than half a century ago as a hypothetical microtubule-independent structure that can exert a force on the spindle (Forer, 1965; Goldman and Rebhun, 1969; Pickett-Heaps et al., 1997). Definitions and terminologies of the spindle matrix vary or are loosely used based on the observations in different systems by different research groups (Walker et al., 2000; Tsai et al., 2006; Lince-Faria et al., 2009; Johansen et al., 2011). Spindle matrices observed by different researchers may not necessarily be identical or equivalent structures. Furthermore, a similar term, the spindle envelope, is sometimes used interchangeably for the spindle matrix, or sometimes for a similar but different structure that surrounds the spindle (Civelekoglu-Scholey et al., 2010; Zheng, 2010; Schweizer et al., 2015). As they have not been studied in the same cell, their relationship is unclear. In this report, we use the terms, the spindle matrix and the spindle envelope, purely by their microtubule-independent localization without functional implications. The spindle matrix is a structure or substance that localizes to the spindle and broader areas of the spindle, while the spindle envelope is one that surrounds the spindle without concentration on the spindle itself.

Multiple hypotheses have been proposed for roles of the spindle matrix or envelope (Lee and Wadsworth, 2009; Johansen and Johansen, 2009; Zheng, 2010; Johansen et al., 2011; Schweizer et al., 2014; Woodruff, 2018; Huang et al., 2018).

For example, it may act as a barrier or substance that accumulates or excludes some molecules or organelles to promote the spindle assembly/stability (Schweizer et al., 2015). Alternatively, it may act as an elastic envelope or substance to mechanically contribute to the force balance within the spindle (Civelekoglu-Scholey et al., 2010). So far, there is no definitive proof to support or exclude a certain hypothesis, and it may well have multiple functions, or have different functions in different systems.

Some molecular studies of the spindle matrix have been carried out in *Xenopus* egg extract and in *Drosophila* mitotic cells. In *Xenopus* egg extract, the lamin B matrix was found to be associated with spindles formed around Aurora A beads. Its association depends on Ran-GTP but not microtubules. It contains other proteins important for spindle assembly, such as NuMA, kinesin-5 Eg5, XMAP215, and their association with the matrix depends on lamin B but not microtubules (Tsai et al., 2006). The conserved protein BuGZ was also found to be associated with the lamin B matrix independently of microtubules and important for the association of the other spindle-associated proteins with the lamin B matrix (Tsai et al., 2006). BuGZ itself can phase-separate and bind to microtubules (Jiang et al., 2015). However, BuGZ does not show spindle localization in mouse oocytes (So et al., 2019).

In *Drosophila* mitotic cells, four proteins have so far been identified as spindle matrix components, namely, Skeletor (Skel), Chromator (Chro), Megator (Mgtor), and Enhanced adult sensory threshold (East) (Walker et al., 2000; Rath et al., 2004; Qi et al., 2004; Qi et al., 2005). All of them localize to the nucleus during interphase, although they are not all in the same nuclear compartment (Johansen and Johansen, 2007). Skel and Mgtor were shown to maintain a spindle-like structure after microtubule depolymerization (Walker et al., 2000; Qi et al., 2004). Chro was shown to directly interact with microtubules (Yao et al., 2014). Some of the proteins (Skel-Chro, Mgtor-East) were shown to physically interact with each other (Rath et al., 2004; Qi et al., 2005), although localization dependencies among these proteins were not reported. Loss of these proteins caused different abnormalities in microtubule assembly, chromosome dynamics, and the spindle assembly checkpoint (Johansen and Johansen, 2009). Separately, in *Drosophila* oocytes, Aberrant X segregation (Axs) with a transmembrane domain surrounds the meiotic spindle. An *Axs* dominant mutation results in high frequencies of shorter spindles, chromosome misalignment, and achiasmatic chromosome nondisjunction (Kramer and Hawley, 2003).

So far, only limited studies have been carried out on the spindle matrix or the spindle envelope, which remain enigmatic. Here, we report identification of Slender lobes (Sle) as an integral component of the spindle matrix and the conserved kinase Greatwall (Gwl) as a spindle envelope protein in *Drosophila* oocytes. We found that Gwl is important for assembly of Sle into the spindle matrix, which in turn determines the location of the Gwl spindle envelope. We further show that Sle is important for the structure and function of the meiotic spindle in oocytes.

## Results

### Sle is a component of the spindle matrix in meiotic oocytes

Previously, we have identified microtubule-associated proteins regulated by the phospho-docking protein 14-3-3 from *Drosophila* ovary extract (Repton et al., 2022). By GFP tagging, we found that one of them, the Sle protein, showed spindle-like localization in mature oocytes, which undergo programmed arrest at meiotic metaphase I. *sle* was originally identified as a mutant defective in brain development in *Drosophila*, and shown to encode a nucleolar protein lacking recognizable functional domains (Orihara-Ono et al., 2005). This study also reported its localization to the mitotic spindle without further analysis.

**Figure S1.**
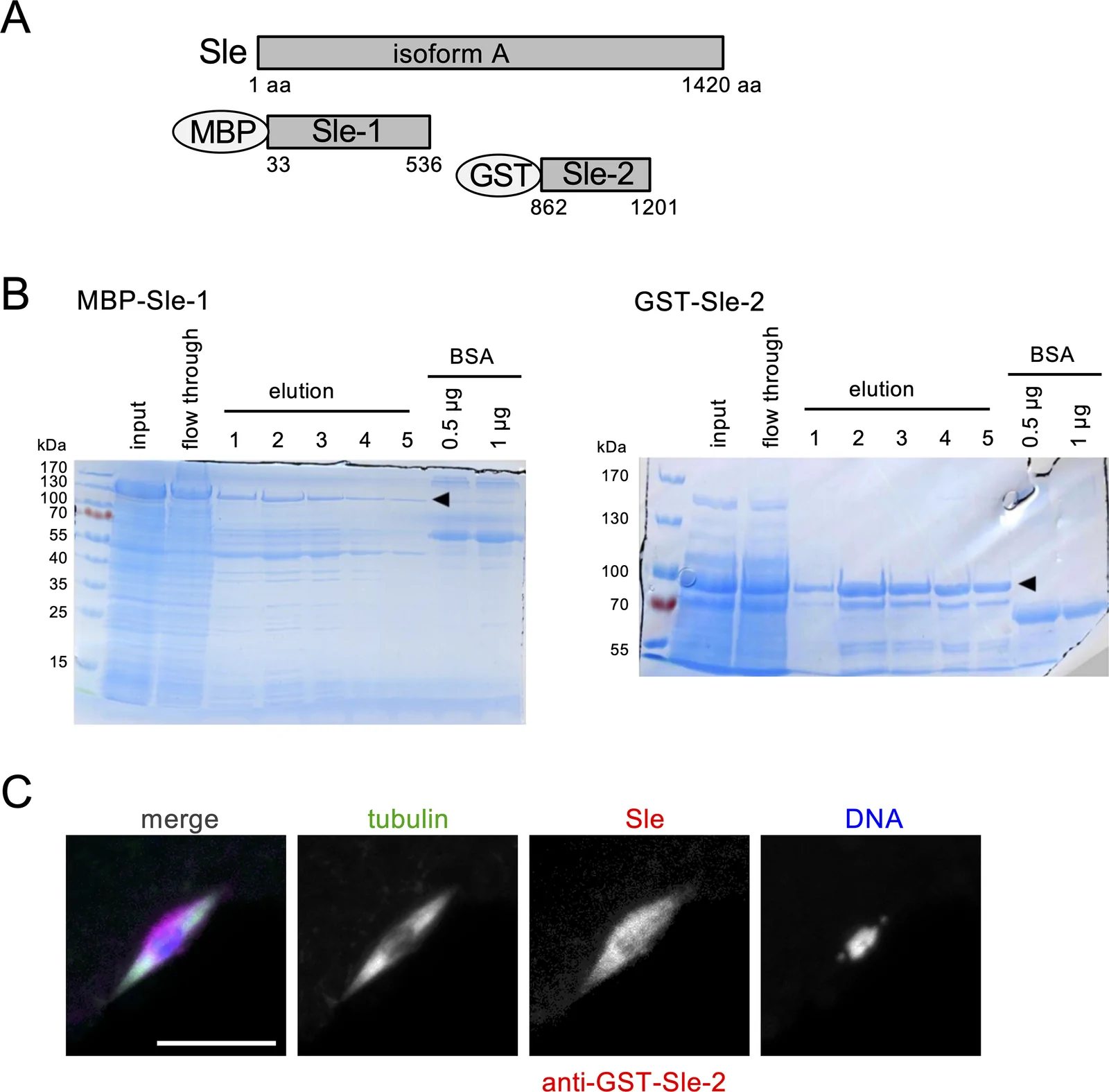
**Sle antibodies. (A)** Regions of Sle used as antigens to generate anti-Sle antibodies. **(B)** Production and purification of Sle fragments from bacteria. Soluble fraction (Input) containing MBP-Sle-1 or GST-Sle-2 was run through a column with amylose resin. The flow-through and five eluted fractions were run on a protein gel together with BSA at known amounts for quantification. The arrowheads indicate MBP-Sle-1 or GST-Sle-2. **(C)** Immunostaining of a metaphase I oocyte using antibodies against GST-Sle-2 and α-tubulin. Bar = 10 μm. Source data are available for this figure: SourceData FS1.

**Figure 1.**
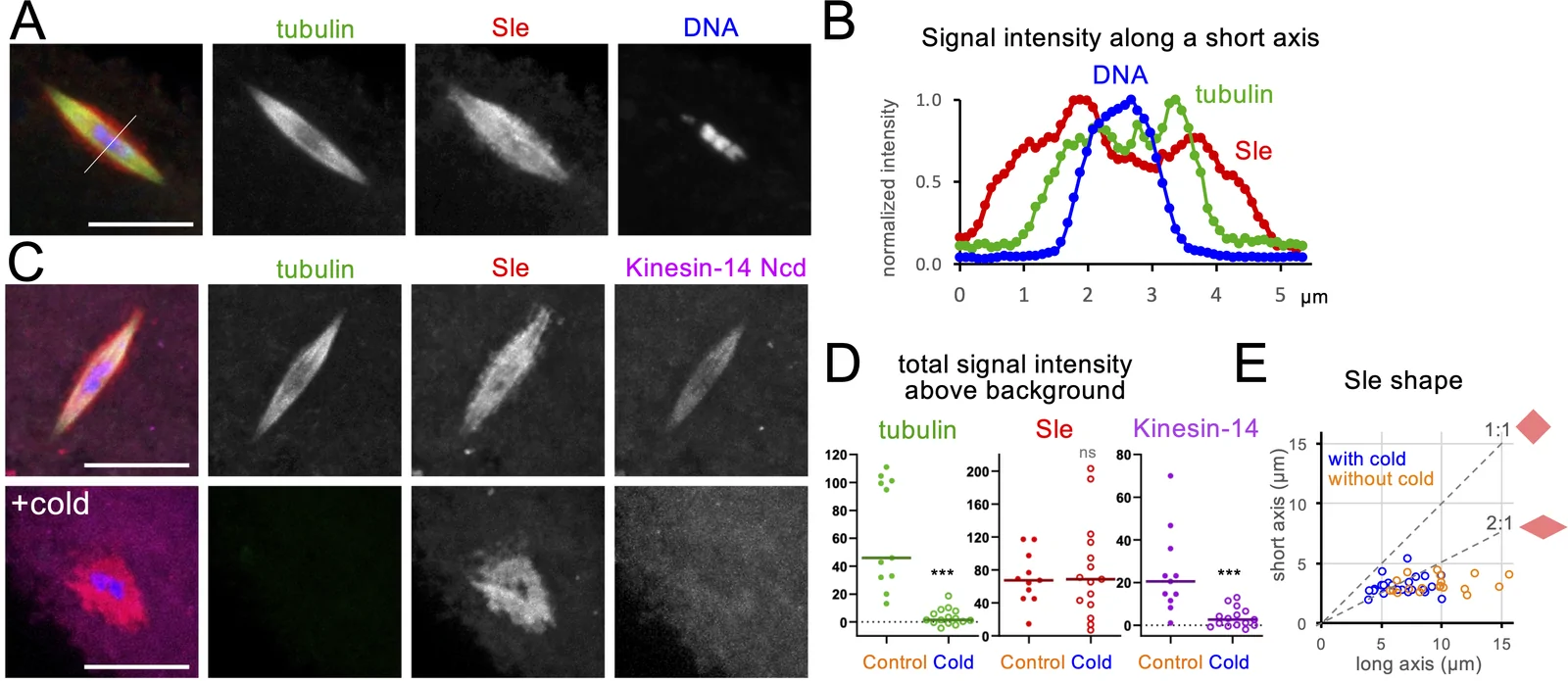
**Sle is a component of the spindle matrix in oocyte meiosis. (A)** Sle localization in oocyte meiosis. Mature oocytes, which arrest in metaphase I, were immunostained with antibodies against Sle and α-tubulin. Sle localized wider than the spindle. Scale bar = 10 μm. **(B)** Signal intensity along the line drawn in A on the short axis of the spindle. They are normalized against the maximum intensity in each channel. **(C)** Metaphase I oocytes with or without cold treatment were immunostained with antibodies against Sle, kinesin-14 Ncd, and α-tubulin. After microtubules were depolymerized by cold treatment, α-tubulin and kinesin-14 Ncd were diffused, while Sle occupied at an elongated area surrounds the chromosomes. Scale bar = 10 μm. **(D)** Total signal intensities in the spindle area above the ooplasm in oocytes with or without cold treatment following normalization using the mean background. The intensities of α-tubulin and kinesin-14 Ncd were greatly reduced nearly to the ooplasm levels by cold treatment (P = 0.00000052, 0.000070; U test), while the intensity of Sle did not significantly change (P = 1.0). ***P < 0.001. ns, P > 0.05. *n* = 11, 15 (control, cold). **(E)** Shape of the area occupied by Sle. The long and short axes of Sle with or without cold treatment were measured and plotted. The long axis of the Sle area was shortened after microtubule depolymerization by cold treatment, but the area was still elongated.

To determine the localization of the endogenous Sle protein relative to the spindle microtubules, we generated antibodies against two nonoverlapping regions of Sle (Fig. S1) and used them to immunostain mature oocytes together with an α-tubulin antibody. Both of the Sle antibodies showed that Sle localization largely overlaps with the spindle microtubules, and strikingly that the localization was often broader (by a median of 0.53 μm) than the spindle defined by tubulin signals (Fig. 1, A and B; and Fig. 7 I; and Fig. S1).

To test whether the Sle localization depends on microtubules, we depolymerized microtubules by cold treatment and then immunostained oocytes for α-tubulin, Sle, and kinesin-14 Ncd. Kinesin-14 Ncd was used as a control representing a conventional microtubule-associated protein. After cold treatment, spindle microtubules were depolymerized and kinesin-14 Ncd was diffused, while Sle still occupied an area around the chromosomes (Fig. 1 C). The total signal intensity of Sle was not significantly reduced, while α-tubulin and kinesin-14/Ncd signal intensities dropped to the background levels (Fig. 1 D). The area occupied by Sle was still elongated, although the long axis was shortened (Fig. 1 E).

Therefore, Sle forms or associates with a structure/substance that can be maintained independently of microtubules, which we refer to here as the Sle spindle matrix.

### Sle localizes to the nucleus in meiotic prophase, but is reassembled to the spindle after nuclear envelope breakdown in oocytes

**Figure 2.**
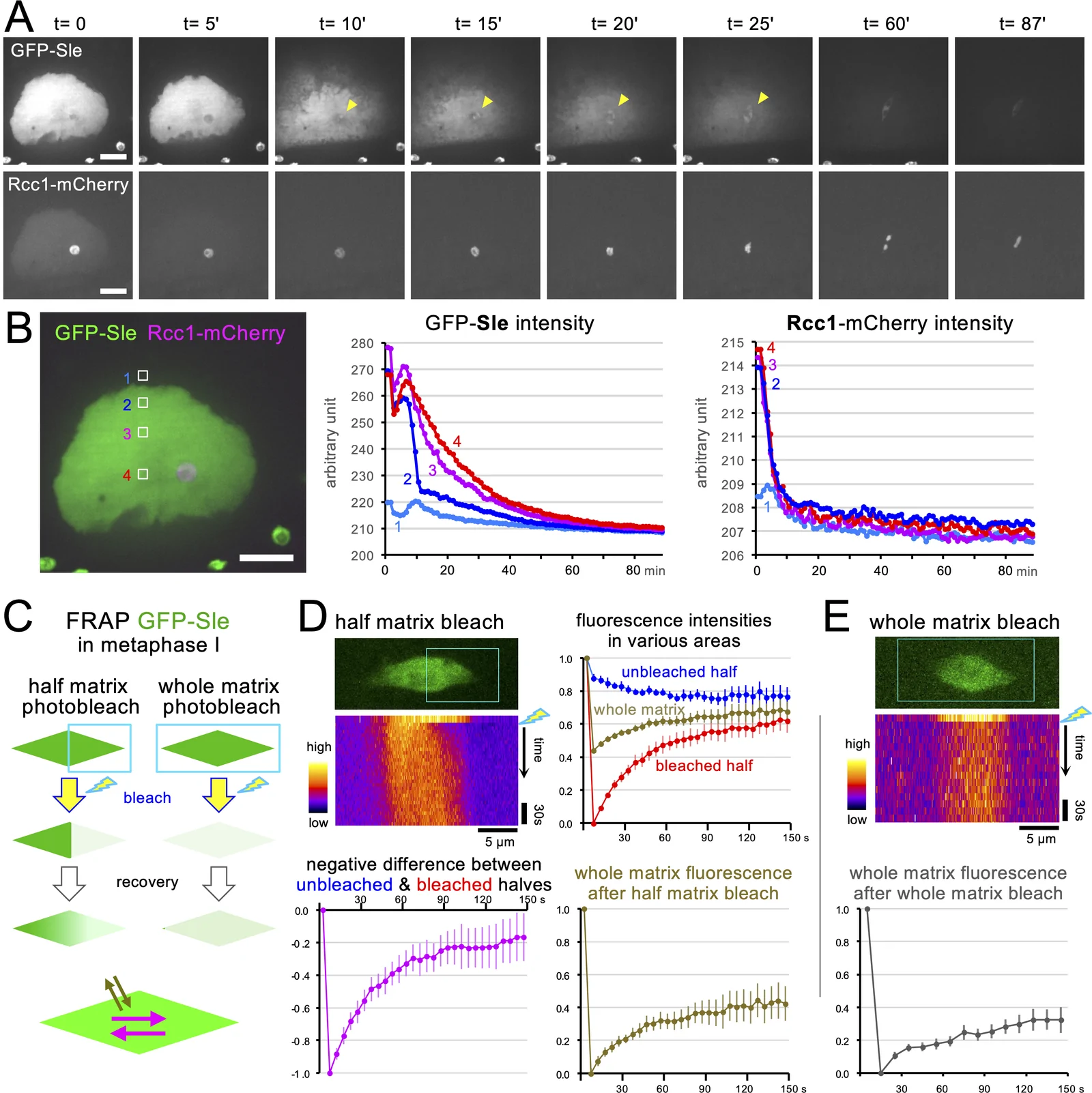
**Sle is actively recruited to or selectively stabilized on the spindle matrix. (A)** Time-lapse live images from NEBD in oocytes expressing GFP-Sle and Rcc1-mCherry. Rcc1-mCherry is a marker of the meiotic chromosomes, which form a compact cluster before NEBD. The arrowheads indicate accumulation of GFP-Sle near the chromosomes. This accumulation started 10 min after NEBD, which roughly coincides with initiation of the spindle formation. Scale bars = 10 μm. **(B)** Changes of the signal intensities in various regions (1–4) of the oocytes. Rcc1-mCherry in the nucleoplasm diffused quickly, but GFP-Sle diffused more slowly. Scale bar = 10 μm. **(C–E)** FRAP analysis of GFP-Sle in metaphase I oocytes. GFP-Sle in a half of the spindle matrix (D) or in the whole spindle matrix (E) was photobleached and followed over time. The analysis revealed predominant exchange within the spindle matrix and slow exchange with the ooplasm, which are shown by the recovery curves of differences between unbleached and bleached halves and the whole matrix fluorescence, respectively. **(D)** FRAP of a half spindle matrix. The bleached area is marked by a blue box on a prebleach image. In the kymograph below, fluorescence intensities are shown against time using the color scheme on the left after maximum intensity projection onto the long spindle axis. It shows gradual spread of GFP-Sle from the unbleached half of the spindle matrix. In the top right graph, mean fluorescence intensities of various areas are normalized against the pre- and postbleach values in the bleached half. The lower left graph shows the negative differences of the normalized intensities between the unbleached half and the bleached half. The lower right graph shows intensities of the whole spindle matrix. Each of them is normalized against the pre- and postbleach values. The error bars indicate the standard errors of the mean. *n* = 10. **(E)** FRAP of the whole spindle matrix. The bleached area is marked by a blue box on a prebleach image. In the kymograph below, fluorescence intensities are shown against time using the color scheme on the left after maximum intensity projection onto the long spindle axis. The graph shows normalized fluorescence intensities in a whole spindle area over time. Error bars indicate the standard errors of the mean. *n* = 7.

**Figure 3.**
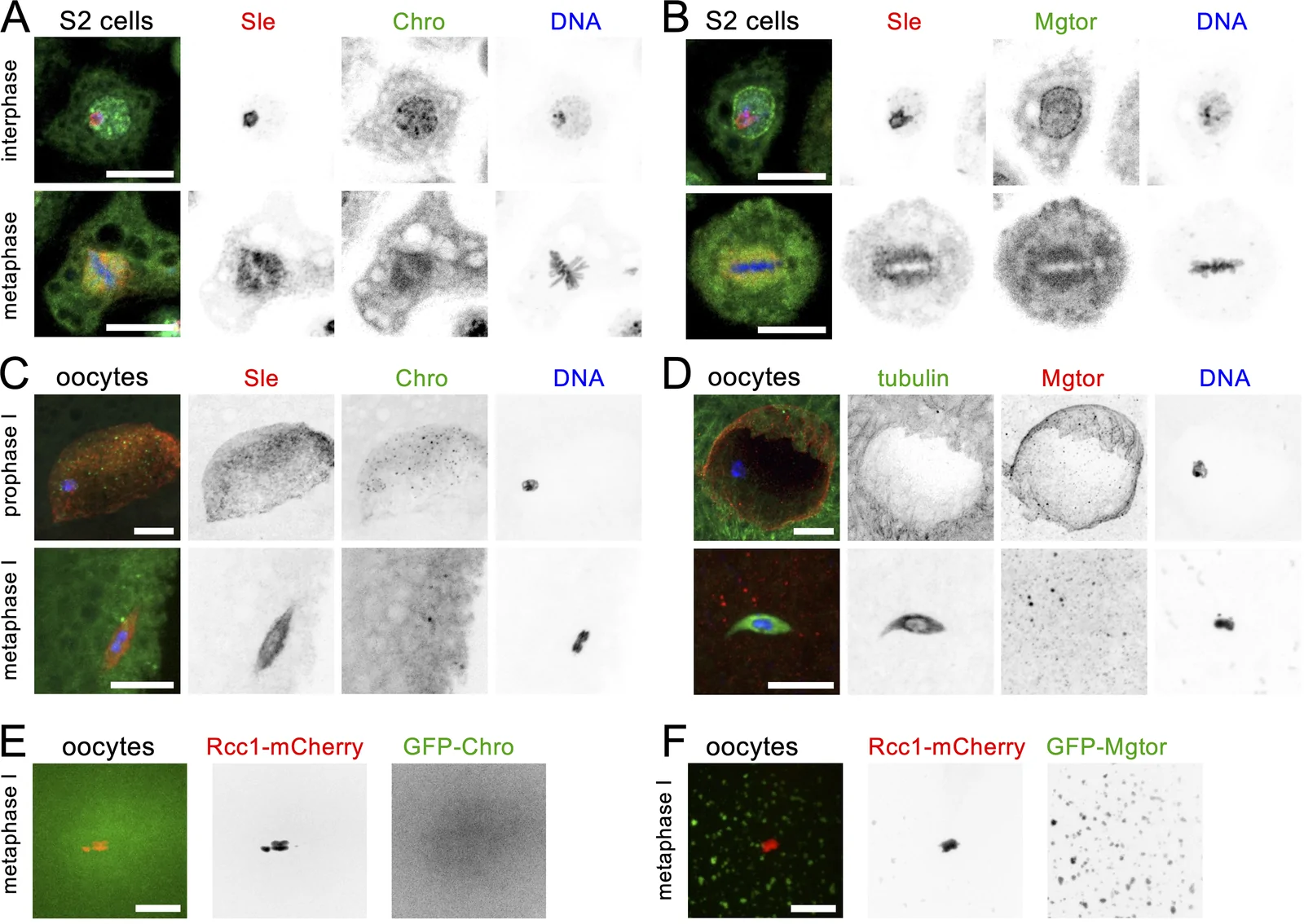
**Composition of the spindle matrix is different in oocytes from mitotic cells.** Higher signal intensity is displayed in darker gray except merged images. Intensity quantification is shown in Fig. S2 E. Scale bars = 10 μm. **(A)** Sle localization with the known spindle matrix protein Chro in S2 cells. S2 cells were immunostained with antibodies against Sle and Chro. In interphase, Sle and Chro localized to the nucleolus and the nucleus, respectively, as previously reported (Orihara-Ono et al., 2005; Rath et al., 2004). In mitotic metaphase, Sle and Chro colocalized to the spindle matrix. *n* = 28. **(B)** Sle localization with the known spindle matrix protein Mgtor in S2 cells. S2 cells were immunostained with antibodies against Sle and Mgtor. In interphase, Mgtor localized to the nuclear rim and nucleoplasm as previously reported (Qi et al., 2004). In mitotic metaphase, Sle and Mgtor colocalized to the spindle matrix. *n* = 18. **(C)** Chro localization in oocytes. Oocytes were immunostained with antibodies against Sle and Chro. In prophase I, Sle and Chro localized to the nucleoplasm. In metaphase I, Sle localized to the spindle matrix, while Chro showed diffused localization. *n* = 20. **(D)** Mgtor localization in oocytes. Oocytes were immunostained with antibodies against Mgtor and α-tubulin. In prophase I, Mgtor localized to the nuclear rim. In metaphase I, Mgtor showed diffused localization. *n* = 29. **(E)** Live imaging of a metaphase I oocyte expressing GFP-Chro and Rcc1-mCherry. Rcc1-mCherry highlights chromosomes. GFP-Chro showed diffused localization. Scale bar = 10 μm. *n* = 12. **(F)** Live imaging of a metaphase I oocyte expressing GFP-Mgtor and Rcc1-mCherry. GFP-Mgtor showed diffused localization with some concentrated clusters all over the ooplasm. *n* = 10.

Several components of the spindle matrix have been identified in *Drosophila* mitosis (Walker et al., 2000; Rath et al., 2004; Qi et al., 2004; Qi et al., 2005). All of them localize to the nucleus during interphase, and in mitosis, the spindle matrix forms in the region where the nucleus was previously located. Therefore, the spindle matrix may be just a remnant of the nuclear materials not diffused after nuclear envelope breakdown (NEBD). In meiotic prophase I in oocytes, Sle also localizes to the nucleoplasm (Fig. 2 A and Fig. 3 C). However, the oocyte nucleus is much larger than the meiotic spindle (30–40 μm vs 10–15 μm), suggesting active assembly or stabilization of Sle into the spindle matrix in oocytes. To determine the assembly dynamics, we live-imaged oocytes expressing GFP-Sle and a chromosomal marker (Rcc1-mCherry) (Fig. 2 A).

**Figure S2.**
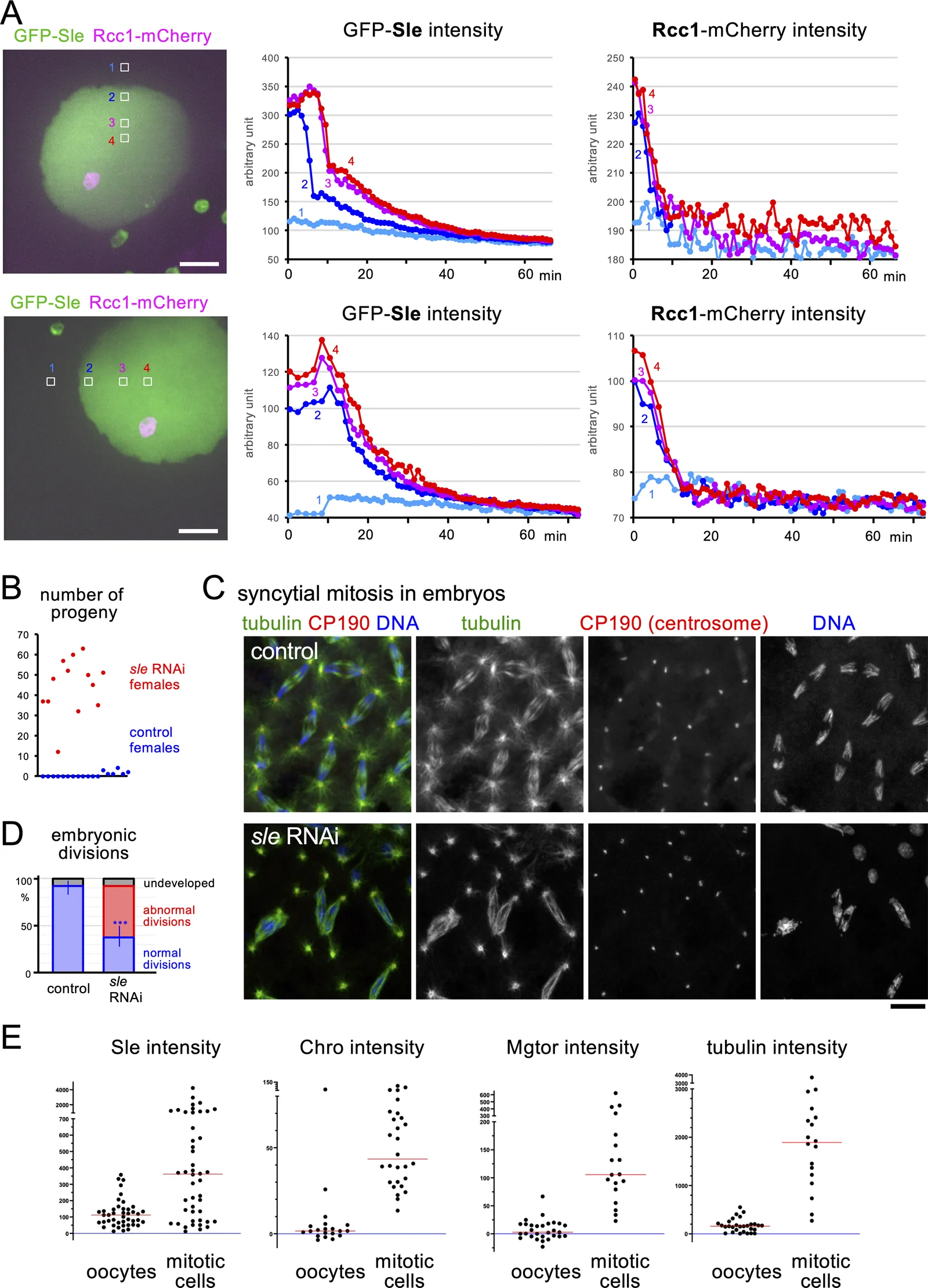
**Sle localization and function in mitotic cells and oocytes. (A)** Changes of the signal intensities in various regions (1–4) from NEBD in oocytes expressing GFP-Sle and Rcc1-mCherry in addition to the oocyte in Fig. 2 A. Rcc1-mCherry in the nucleoplasm diffused quickly, but GFP-Sle diffused more slowly. Scale bar = 10 μm. **(B)** Fertility test of females expressing *sle* shRNA (RNAi 1) in ovaries. The number of progeny from five RNAi females in each vial was counted. **(C)** Syncytial mitosis in early embryos from *sle* RNAi mothers and control (no RNAi) mothers. Eggs from *sle* RNAi females (RNAi 1) were collected for 60 min and further aged for 90 min at 25°C and immunostained with antibodies against α-tubulin and the centrosomal protein CP190. Typical abnormalities in syncytial embryos from *sle* RNAi mothers include asynchronous divisions, free centrosomes, acentrosomal spindles, abnormally shaped spindles, chromosome mis-segregation, and unequal density of nuclei. **(D)** Quantification of early embryonic divisions. “Normal divisions” includes synchronous mitosis with bipolar spindles and centrosomes at the poles, interphase nuclei with a uniform density, and stages beyond cellularization. “Abnormal divisions” includes the abnormalities described in C. “Undeveloped” includes eggs with a few DNA masses. Normal divisions are significantly less frequent in *sle* RNAi embryos than control (P = 0.0001; *n* = 53, 71; Fisher’s exact test). ***P < 0.001. The error bars represent 95% confidence intervals of the frequencies of normal divisions. **(E)** Signal intensities of Sle, Chro, and Mgtor in metaphase I oocytes and mitotic S2 cells. S2 cells and mature oocytes were immunostained (shown in Fig. 3). The graphs show the total signal intensities in the spindle matrix area above the cytoplasm in mitotic S2 cells and metaphase I oocytes following normalization using the background. The horizontal lines represent the medians. *n* = 43, 46, 20, 28, 28, 18, 29, 18.

Before NEBD, GFP-Sle accumulated in the large prophase I nucleus, while Rcc1-mCherry distributed weakly and uniformly in the nucleoplasm with a high concentration on the compactly clustered chromosomes. Once the nucleus started to break down, Rcc1-mCherry in the nucleoplasm diffused quickly within 10 min. In contrast, GFP-Sle appeared to remain more concentrated in the collapsing nucleus for much longer (∼40 min after NEBD) before it fully diffused (Fig. 2 B; Fig. S2). Sle began accumulating around the chromosomes and grew into a spindle-like shape 11.0 ± 1.2 min (mean ± SD, *n* = 4) after the start of the NEBD (Fig. 2 A). This timing roughly corresponds to the assembly of spindle microtubules in oocytes (Colombié et al., 2008; Głuszek et al., 2015; Costa and Ohkura, 2019).

This dynamic suggests that the Sle protein in the prophase nucleus is in a form that is only slowly diffusible. Furthermore, Sle is actively recruited or selectively stabilized on the spindle region after NEBD, rather than simply a remnant of the nuclear Sle.

### Sle in the spindle matrix is dynamic

To test molecular dynamics of Sle, we carried out fluorescence recovery after photobleaching (FRAP) in metaphase I oocytes expressing GFP-Sle (Fig. 2 C). GFP-Sle within a half of the spindle matrix was photobleached, and recovery of the fluorescence was monitored over time (Fig. 2 D). The kymograph showed that fluorescence recovery in the bleached half started and spread from the region closest to the unbleached half. This recovery of fluorescence in the bleached half was compensated mainly by a reduction in the unbleached half. This shows that Sle is dynamically exchanged within the spindle matrix.

To estimate the exchange of Sle between the spindle matrix and the ooplasm, the total fluorescence of the whole spindle matrix including both bleached and unbleached halves was measured over time after photobleaching a half of the spindle matrix. The total fluorescence in the whole spindle matrix slowly recovered, indicating that Sle exchanges predominantly within the spindle matrix but also with the ooplasm (Fig. 2 D). This slow exchange of Sle between the spindle matrix and the ooplasm was further confirmed by FRAP of the whole spindle matrix (Fig. 2 E). These FRAP studies revealed the dynamic nature of Sle in the spindle matrix.

### The composition of the spindle matrix in oocytes differs from mitotic cells

Several components of the spindle matrix have been identified in *Drosophila* mitosis (Walker et al., 2000; Rath et al., 2004; Qi et al., 2004; Qi et al., 2005). To determine whether the composition of the spindle matrix differs between mitotic cells and meiotic oocytes, Sle was first co-immunostained with two known spindle matrix proteins in mitosis, Chro and Mgtor, in the Schneider’s 2 (S2) cell line (Fig. 3, A and B). Sle colocalized with these two proteins in metaphase, showing Sle is a spindle matrix protein also in mitosis (Fig. 3, A and B; and Fig. S2 E). In meiotic prophase I, the same antibodies against Chro and Mgtor gave clear signals in the nucleoplasm and on the nuclear rim, respectively (Fig. 3, C and D), as expected from their localization in mitotic cycles (Fig. 3, A and B; Qi et al., 2004; Rath et al., 2004). In contrast, in metaphase I oocytes, neither gave clear signal concentration on or around the spindle (Fig. 3, C and D; and Fig. S2 E). To exclude a possibility of technical issues during immunostaining, we live-imaged oocytes expressing GFP-Chro or GFP-Mgtor together with the chromosomal marker Rcc1-mCherry. We did not observe detectable spindle-like signals in either case (Fig. 3, E and F). These results showed that the spindle matrix in oocytes has a different composition from that in mitotic cells, but Sle is a component of the spindle matrix in both cell types.

### Sle is important for the spindle architecture in oocytes

**Figure 4.**
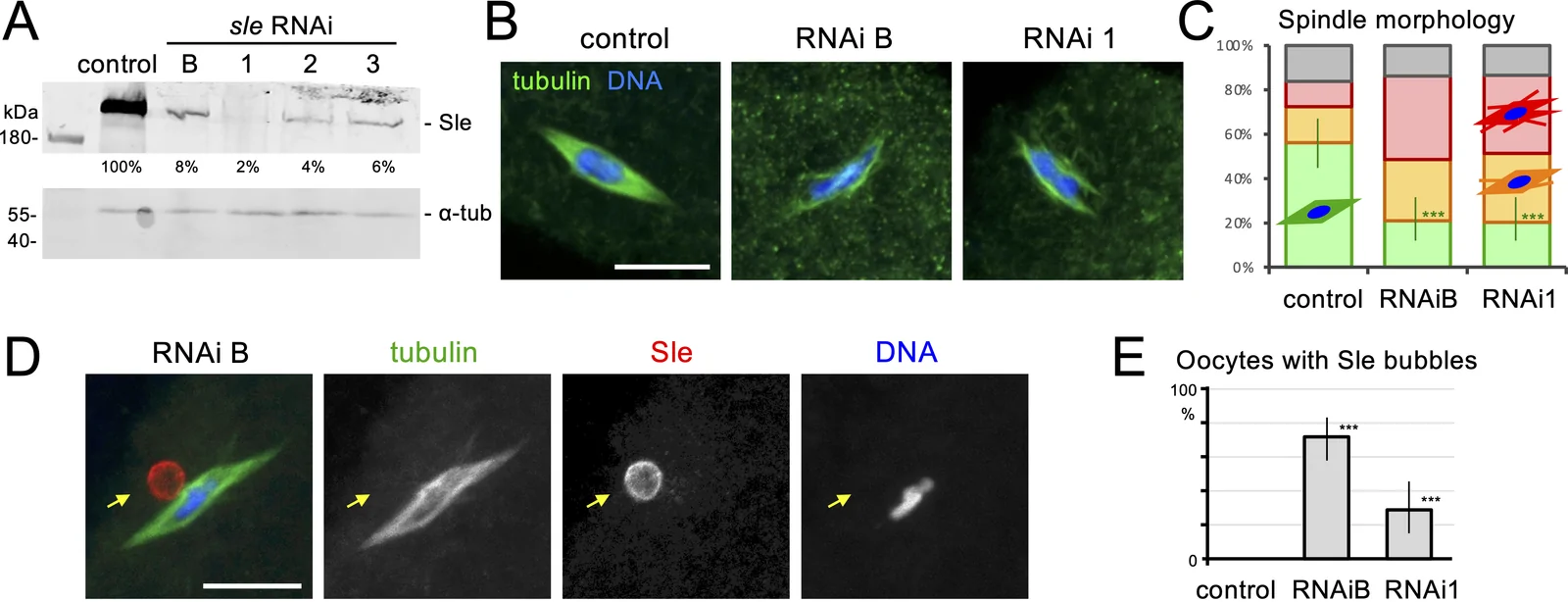
**Sle is important for spindle organization in oocytes. (A)** Western blot of ovaries from various *sle* RNAi lines probed with Sle and α-tubulin antibodies. The line B was obtained from Bloomington *Drosophila* Stock Center, while 1, 2, and 3 were generated for this study. shRNA against *sle* was expressed in ovaries after completion of recombination. Band intensities above the background are shown in percentages relative to the control. **(B)** Metaphase I spindles in control and *sle*-silenced oocytes. Oocytes were immunostained with Sle and α-tubulin antibodies. Spindles were generally bipolar but most have microtubule bundles branching out and/or split poles in *sle*-silenced oocytes. Scale bar = 10 μm. **(C)** Morphologies of the metaphase I spindle in control and *sle*-silenced oocytes. From the bottom to the top: (green) smooth bipolar spindles with focused poles, (orange) bipolar spindles with thin microtubule bundles branching out from the sides, (red) bipolar spindles with either split poles, or strong or many thin microtubule bundles branching out, and (gray) spindles with other morphologies including a nonbipolar spindles and multiple spindles. Note that meiotic spindles dynamically change their morphologies even during metaphase I arrest in wild type. The error bars represent 95% confidence intervals. P = 0.000012, 0.0000062 (frequencies of smooth bipolar spindles with focused poles compared with the control; Fisher’s exact test). ***P < 0.001. *n* = 80, 72, 74. **(D)** Sle bubbles in *sle* RNAi oocytes. *sle*-silenced oocytes (RNAi B) at metaphase I were immunostained with Sle and α-tubulin antibody. Sle was lost from the spindle, but showed bubble-like localization near the spindle. The arrows indicate the position of a Sle bubble. The brightness of Sle is enhanced. Scale bar = 10 μm. **(E)** Frequencies of oocytes with Sle bubbles from the no-RNAi control, modestly depleted RNAi B, and the most depleted RNAi 1. The error bars represent 95% confidence intervals. P = 0.000000, 0.00033 (compared with the control; Fisher’s exact test). ***P < 0.001. *n* = 40, 47, 32. Source data are available for this figure: SourceData F4.

To find out the function of Sle in oocytes, we have generated three transgenic lines expressing shRNA against different regions of the *sle* gene, in addition to a line available publicly. We used a maternal α-tubulin driver (V37), which allows expression of shRNA after completion of mitosis and recombination in female germlines. A western blot showed that all 4 RNAi lines successfully depleted over 90% of the Sle protein in ovaries (Fig. 4 A).

*sle* null mutant females were reported to have underdeveloped ovaries (Orihara-Ono et al., 2005). We found that females from all of the *sle* RNAi lines produced mature oocytes, allowing us to study the role of Sle beyond early oogenesis. The females are nearly infertile and lay eggs that show defects in syncytial mitotic divisions (Fig. S2, B–D).

To define the role of Sle in meiotic spindle formation, we immunostained mature oocytes from the most depleted line (RNAi 1) and modestly depleted line (RNAi B), together with a control without RNAi (Fig. 4, B and C). In the control, most meiotic spindles had a bipolar spindle with focused poles. In both RNAi lines, most spindles had bipolar shapes with split poles or microtubule bundles branching out from the sides of the spindle. Therefore, Sle is important for the proper architecture of the metaphase I spindle in oocytes.

When Sle was depleted, Sle signal was lost from the spindle, but interestingly showed “bubble-like” structures near the meiotic spindle (Fig. 4 D). The Sle signal intensity inside of the bubbles was much lower than at the rim. Sle bubbles were mostly spherical, but some were elongated. The frequency of oocytes with Sle bubbles was high (72%) in the modestly depleted line, and low (28%) in the most depleted line (Fig. 4 E). In control oocytes without RNAi, such structures were not observed even when signal brightness was enhanced. Therefore, Sle behaves in a dose-dependent manner: at high levels, it forms or localizes to the spindle matrix; at low levels, it instead forms or localizes to a bubble-like structure.

### Sle buffers the effects of compromised kinetochore–microtubule interaction

**Figure 5.**
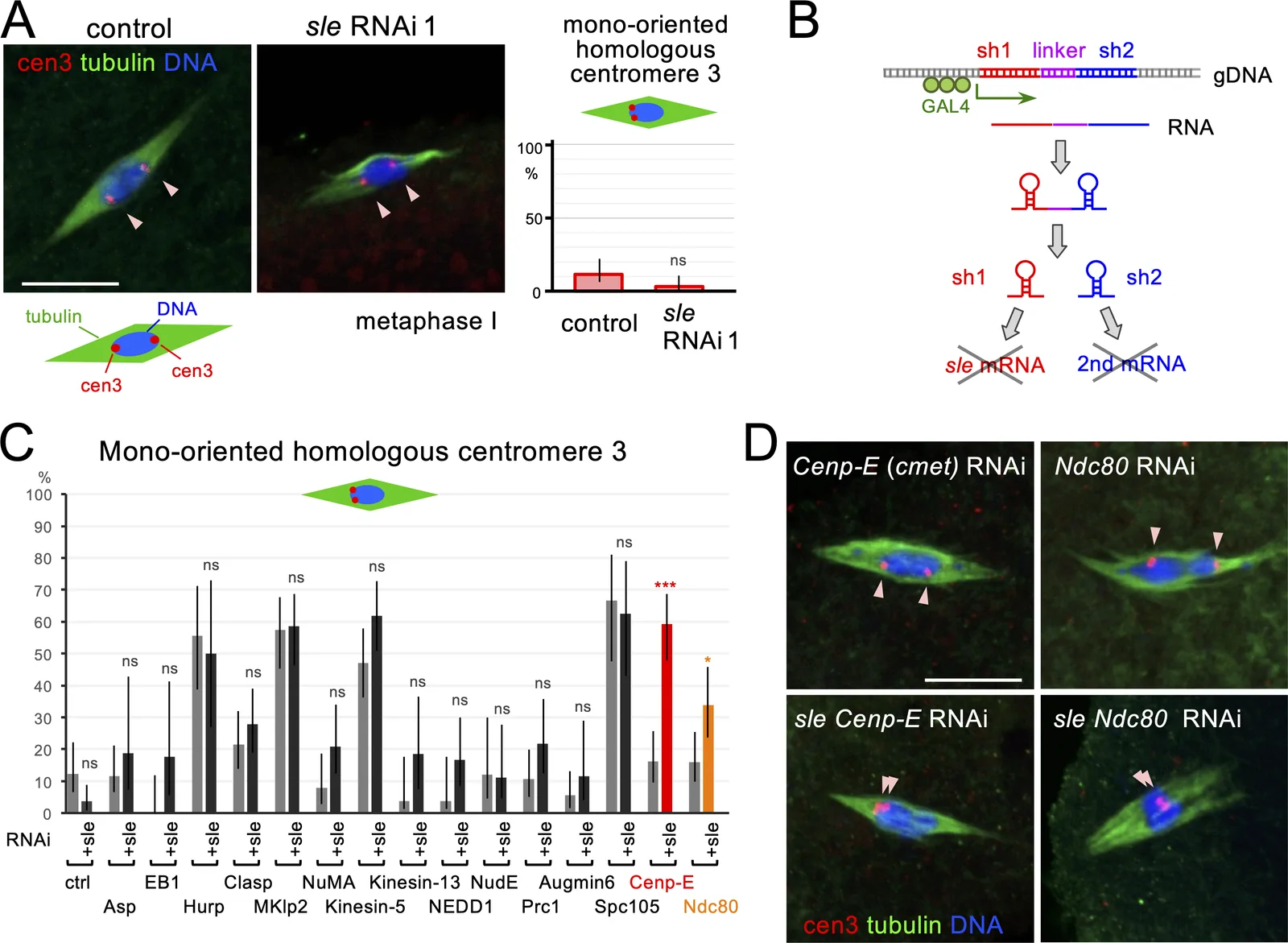
**Cosilencing of *sle* enhanced mono-orientation defects of *Cenp-E/cmet* or *Ndc80* silencing. (A)** Control and *sle* RNAi 1 metaphase I oocytes subjected to *in situ* hybridization probed with pericentromeric dodecasatellite specific to chromosome 3 and immunostaining with α-tubulin. In metaphase I, sister centromeres are attached and form one dot. Mono-oriented homologous centromere 3 (two dots were located closely or in the same half of the chromosome mass along the long axis of the spindle) was infrequent both in control and in *sle* RNAi. P = 0.063 (Fisher’s exact test). ns, P > 0.05. *n* = 65, 81. Scale bar = 10 μm. **(B)** Diagram of double RNAi. GAL4 drives transcription of RNA containing two short hairpins, which silence expression of *sle* and another gene. **(C)** Frequencies of mono-oriented homologous centromere 3 in metaphase I oocytes with silencing of a single gene alone or cosilencing of the gene and *sle* together (+sle; RNAi 1). Common names of the orthologs are used to indicate silenced *Drosophila* genes. Hurp = Mars, MKlp2 = Sub, NuMA = Mud, kinesin-5 = Klp61F, kinesin-13 = Klp10A, NEDD1 = Grip71, Prc1 = Feo, Augmin6 = Dgt6, Spc105 = Spc105R, Cenp-E = Cenp-meta (Cmet). Frequencies of mono-oriented homologous centromere 3 were not significantly different between single silencing and cosilencing with *sle*, except Cenp-E and Ndc80. The error bars represent 95% confidence intervals. P = 0.063, 0.42, 0.051, 0.76, 0.36, 1.0, 0.080. 0.081, 0.19, 0.14, 1, 0.12, 0.28, 0.78, 0.000001, 0.019 (between single and double RNAi; Fisher’s exact test). ***P < 0.001. *P < 0.05. ns, P > 0.05. *n* = 65, 81, 78, 16, 27, 17, 36, 14, 79, 79, 75, 70, 50, 48, 85, 76, 27, 27, 27, 48, 25, 27, 66, 46, 72, 26, 27, 24, 80, 81, 81, 65. **(D)** Metaphase I oocytes subjected to *in situ* hybridization probed with pericentromeric dodecasatellite specific to chromosome 3 and immunostaining with α-tubulin. The arrowheads indicate centromere 3 signals. Scale bar = 10 μm.

The above results showed that Sle is important for spindle architecture. To determine the role of Sle in spindle function, we visualized positions of centromere 3 in metaphase I by fluorescence *in situ* hybridization (FISH). In control metaphase I, each pair of sister centromeres stayed attached, but homologous centromeres were pulled apart toward opposite poles (bi-oriented; Fig. 5 A). *sle* silencing did not increase the frequency of homologous centromeres that located closely or on one side of the chromosome mass (mono-oriented; Fig. 5A). Therefore, Sle appears dispensable for separation of homologous centromeres in metaphase I. Sle is important for normal spindle structure, but may play no role in spindle function to bi-orient or separate centromeres. Alternatively, a Sle function may be hidden by another protein/pathway.

To reveal a function potentially hidden by another protein/pathway, *sle* was cosilenced together with other genes (Fig. 5 B). We selected genes that have known or possible functions in spindle structure or chromosome segregation. FISH of the centromere 3 was used to compare mature oocytes expressing shRNA against one of the genes with oocytes co-expressing shRNAs against *sle* and this gene (Fig. 5 C). In most cases, cosilencing of *sle* did not significantly increase the frequency of mono-oriented centromeres compared with single silencing. In contrast, cosilencing of *sle* dramatically increased the frequency of mono-oriented centromeres caused by silencing of *Cenp-meta (cmet)* from 16% to 59%, and also significantly increased the frequency caused by silencing of *Ndc80* from 16% to 34% (Fig. 5, C and D). Both encode kinetochore proteins, but Cmet (the *Drosophila* Cenp-E ortholog) mediates lateral interaction with microtubules, while Ndc80 mediates end-on attachment (Radford et al., 2015). This suggests that the role of Sle may not be specific to a particular kinetochore protein, but may be more general to ensure proper spindle–chromosome interaction. Spc105R is also a kinetochore protein, but mono-orientation in single *spc105R* RNAi may be already too frequent to see an enhancement. In summary, although Sle is dispensable for bi-orienting homologous centromeres, it becomes crucial when kinetochore–microtubule interaction is compromised. This suggests that the Sle spindle matrix may act as a safety net rather than as an indispensable structure. Further studies are required to clarify the precise role of the Sle spindle matrix.

### The Sle spindle matrix is tightly surrounded by the spindle envelope containing Gwl kinase

**Figure 6.**
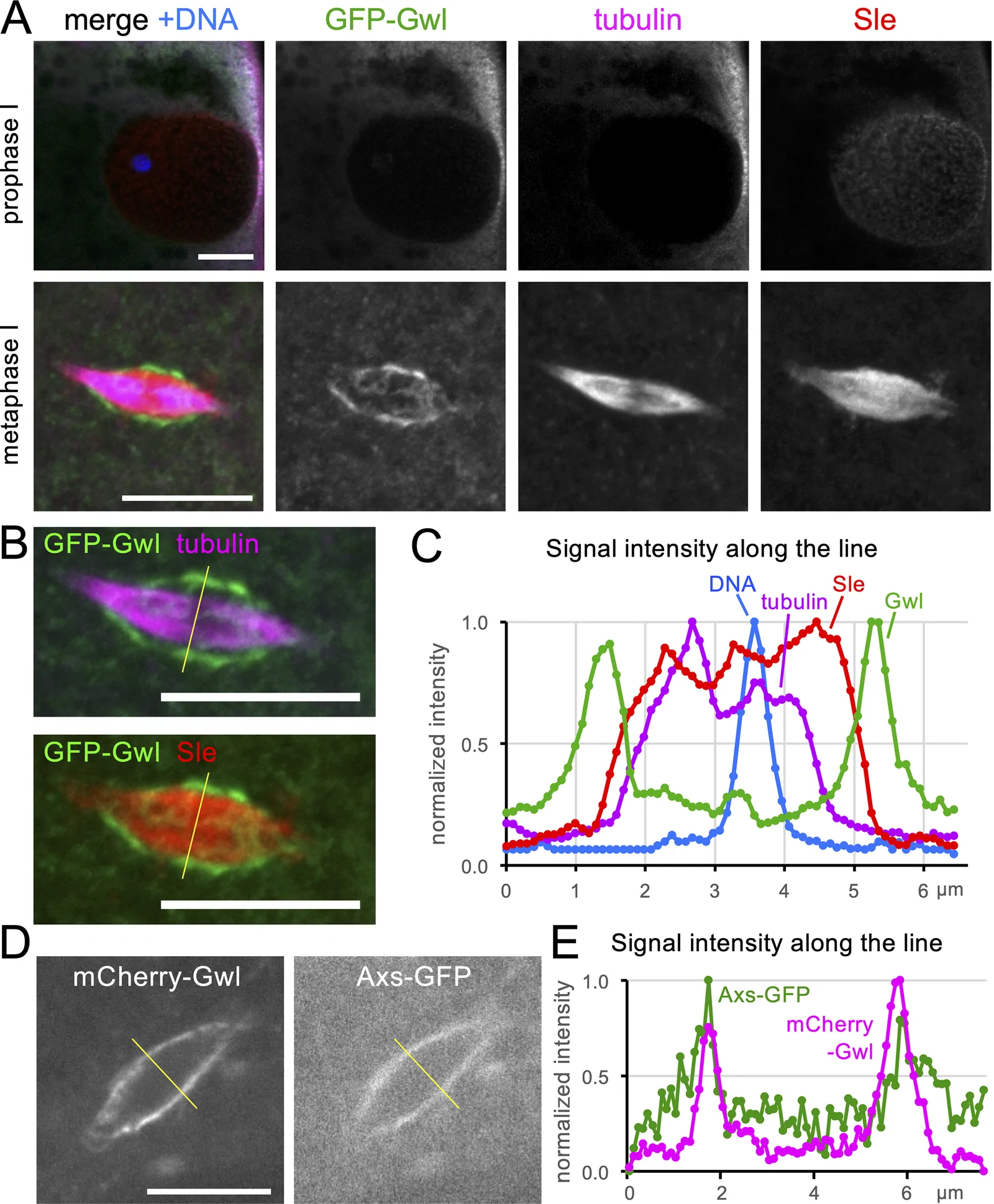
**Gwl kinase localizes to the spindle envelope. (A)** Localization of Gwl in oocytes. Oocytes expressing GFP-Gwl were immunostained with antibodies against GFP and α-tubulin. GFP-Gwl surrounds the metaphase I spindle. Scale bars = 10 μm. **(B)** Magnified merged images of GFP-Gwl and α-tubulin or Sle. GFP-Gwl tightly surrounds Sle, while there are gaps between GFP-Gwl and the spindle microtubules. Scale bars = 10 μm. **(C)** Signal intensities along the line drawn in B on the short axis of the spindle. They are normalized against the maximum intensity of each channel. **(D)** Colocalization of Axs-GFP and mCherry-Gwl expressed in metaphase I oocytes. Oocytes co-expressing Axs-GFP and mCherry-Gwl were live-imaged. A single Z-plane is shown. *n* = 6. Scale bars = 10 μm. **(E)** Signal intensities along the line drawn in D on the short axis of the spindle. They are normalized against the maximum intensity of each channel.

**Figure S3.**
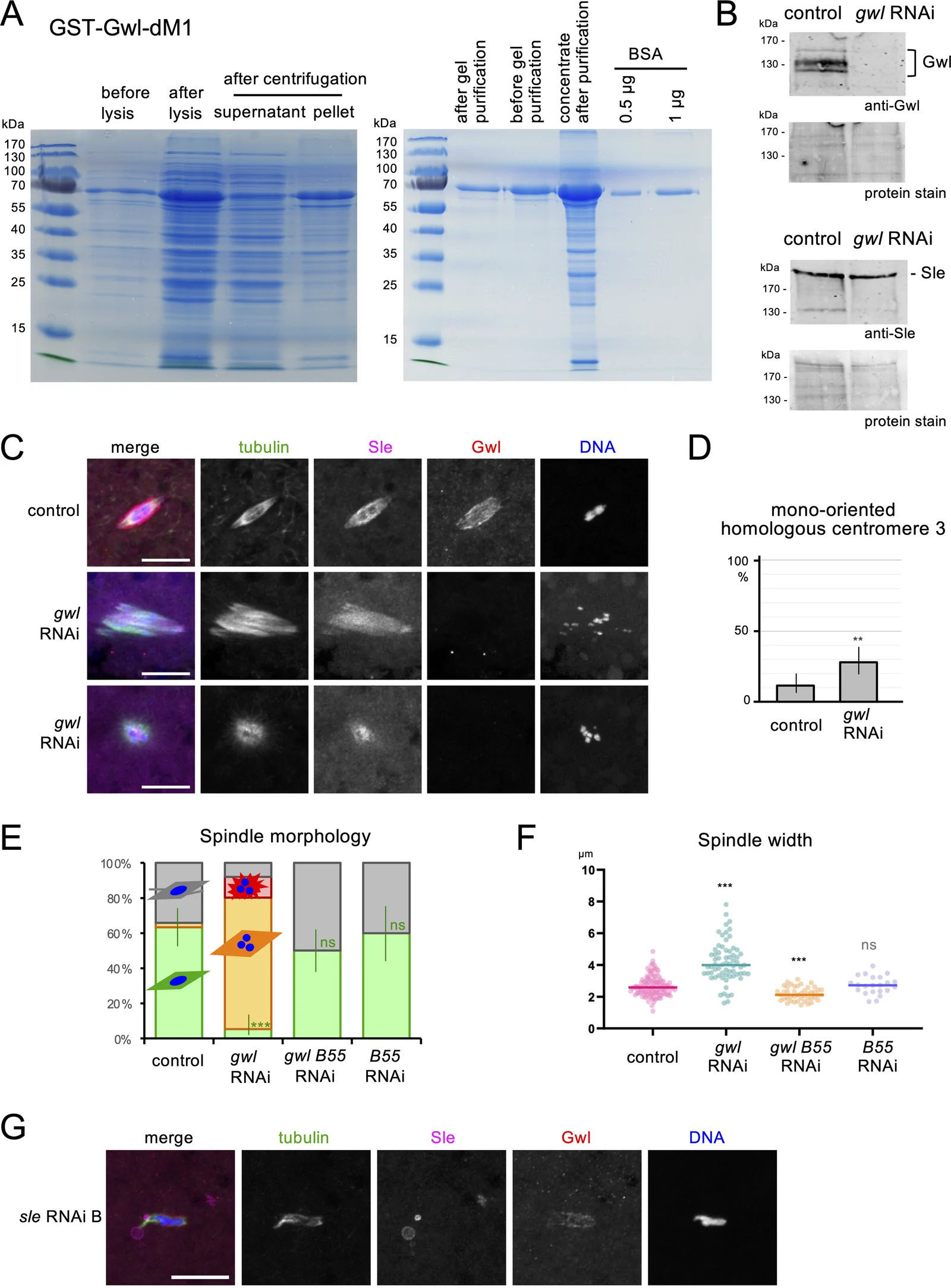
**Gwl antibody, function and localization. (A)** Production and purification of a Gwl fragment from bacteria for antibody production. Most GST-Gwl lacking 224–640 residues (dM1) was found in the insoluble fraction (pellet). Samples from various steps of purification were run together with BSA at known amounts for quantification. **(B)** Western blots of ovaries expressing shRNA against *gwl* (*gwl* RNAi) and control (no RNAi) using the anti-Gwl or anti-Sle antibody, and protein staining of the same membranes as a loading and blotting control. **(C)** Spindle morphologies in control and *gwl* RNAi oocytes in metaphase I. Oocytes were immunostained with antibodies against α-tubulin, Sle, and Gwl. Scale bars = 10 μm. **(D)** Control and *gwl* RNAi metaphase I oocytes subjected to *in situ* hybridization probed with pericentromeric dodecasatellite specific to chromosome 3 and immunostaining with α-tubulin. Oocytes with mono-oriented homologous centromere 3 were more frequent in *gwl* RNAi than in control. The error bars represent 95% confidence intervals. P = 0.0075 (Fisher’s exact test). **P < 0.01. *n* = 92, 81. **(E)** Spindle morphologies in control, *gwl* RNAi, *gwl PP2A-B55* RNAi, and *PP2A-B55* RNAi oocytes in metaphase I. From the bottom to the top in the graph: normal thin bipolar spindles (green), broader bipolar spindles (orange), apolar spindles (red), and spindles with other abnormalities (including spindles with split poles or with branched microtubule bundles, tripolar spindles, multiple separate spindles; gray). The error bars represent 95% confidence intervals. ***P < 0.001. ns, P > 0.05. P = 0.00000000, 0.12, 0.83 (comparing the frequencies of smooth bipolar spindles with focused poles; Fisher’s exact test). *n* = 76, 76, 64, 35. Scale bar = 10 μm. **(F)** Spindle widths in control, *gwl* RNAi, *gwl PP2A-B55* RNAi, and *PP2A-B55* RNAi oocytes in metaphase I. p = 2.22 × 10^−16^, 0.0000040, 0.44 (compared with the control; U test). ***P < 0.001. ns, P > 0.05. *n* = 133, 109, 45, 22. **(G)** Immunostaining of a metaphase I oocyte with *sle* modestly silenced (RNAi B) using antibodies against α-tubulin, Sle, and Gwl. Gwl did not localize to a Sle bubble. Scale bar = 10 μm. Source data are available for this figure: SourceData FS3.

**Figure 7.**
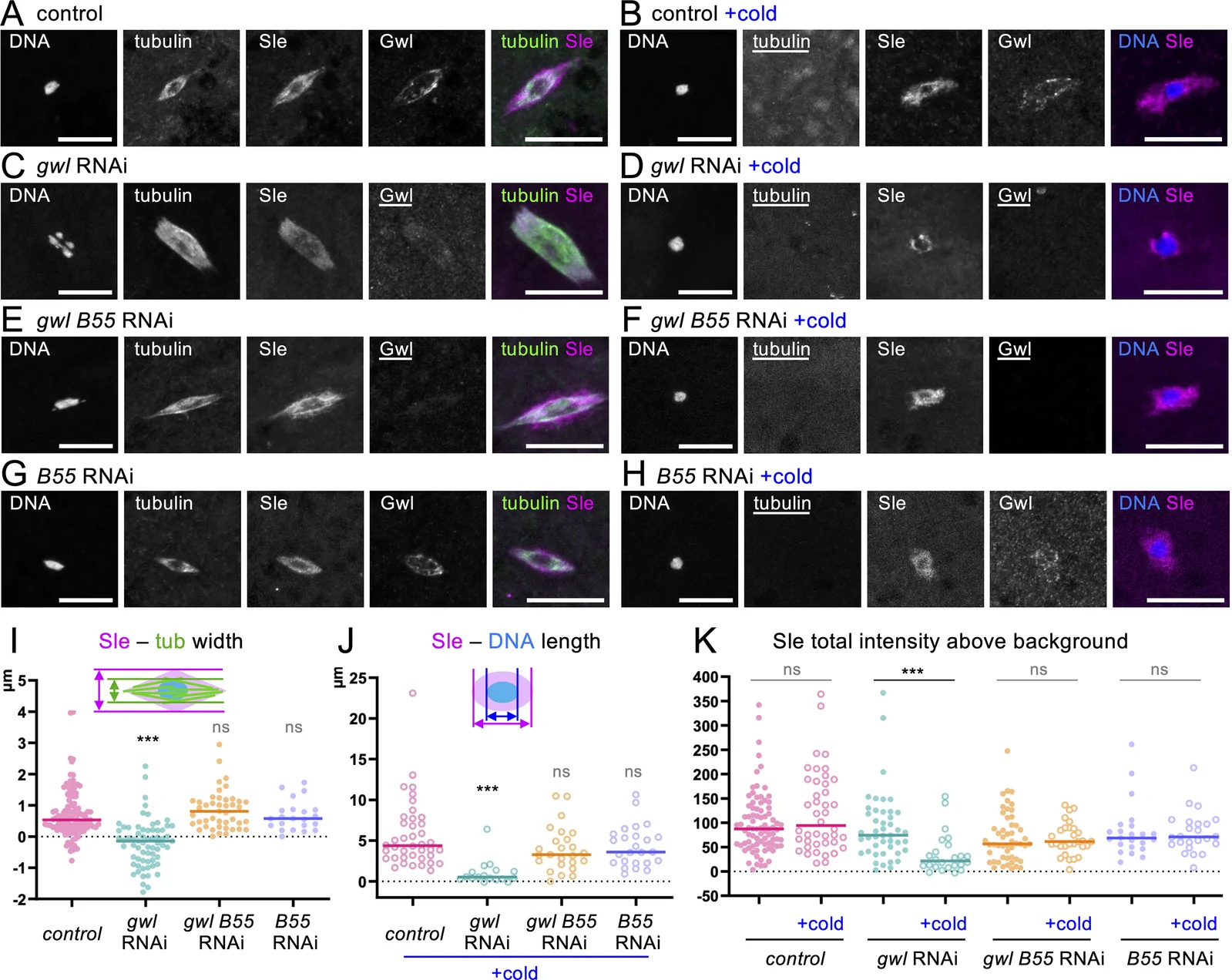
**Spindle envelope kinase Gwl promotes assembly of the Sle spindle matrix through suppression of PP2A-B55. (A–H)** Oocytes from various genotypes and conditions (±cold) were immunostained with antibodies against α-tubulin, Sle, and Gwl. The rightmost panels are merged images with a higher magnification than the others. Underlines indicate channels expected to have weak or diffused signals due to RNAi or depolymerization. Scale bars = 10 μm. **(A)** Control metaphase I oocyte without RNAi. Gwl tightly surrounds Sle, which localizes broader than the spindle. **(B)** Control metaphase I oocyte without RNAi after microtubule depolymerization by cold treatment. Gwl tightly surrounds Sle, which still occupies a large area around the chromosomes. **(C)** Metaphase I oocyte in which *gwl* was silenced. Sle localization mirrored the spindle microtubules on *gwl* silencing, rather than broader localization in the control. **(D)** Metaphase I oocyte in which *gwl* was silenced and microtubules were depolymerized by cold treatment. Sle localized to only a small region around the chromosomes after microtubule depolymerization, rather than to a large area around the chromosomes in the control plus cold (B). **(E)** Metaphase I oocyte in which *PP2A-B55* was cosilenced with *gwl*. Cosilencing of *PP2A-B55* restored a broader Sle localization compared with a *gwl*-silenced oocyte (C). **(F)** Metaphase I oocyte in which *PP2A-B55* was cosilenced with *gwl* and microtubules were depolymerized. Cosilencing of *PP2A-B55* restored Sle localization to a large area around the chromosomes compared with a *gwl*-silenced oocyte (D). **(G)** Metaphase I oocyte in which *PP2A-B55* was silenced. **(H)** Metaphase I oocyte in which *PP2A-B55* was silenced and microtubules were depolymerized by cold treatment. **(I)** Width of the Sle area minus the width of the spindle without cold treatment. The Sle area is wider than the spindle without *gwl* silencing, while *gwl* silencing dramatically reduced the difference. The median is marked with a horizontal line. Cosilencing of *PP2A-B55* restored wider Sle localization. P = 0.63 × 10^−13^, 0.072, 0.77 (compared with the control; U test). ***P < 0.001. ns, P > 0.05. *n* = 114, 67, 45, 22. **(J)** Long axis of the Sle area minus the length of the chromosome mass after microtubule depolymerization by cold treatment. Even after microtubules are depolymerized, the Sle area remains larger than the chromosome mass without RNAi. *gwl* silencing dramatically decreased the length of the Sle area, while cosilencing of *PP2A-B55* rescued this effect of *gwl* silencing. P = 0.00000011, 0.081, 0.22 (compared with the control; U test). ***P < 0.001. ns, P > 0.05. *n* = 40, 17, 25, 25. **(K)** Total intensity of the Sle signal around the chromosomes above the cytoplasmic background after normalization using the mean background. The Sle total intensity did not decrease on *gwl* silencing without microtubule depolymerization by cold, while it dramatically reduced after microtubule depolymerization. P = 0.22, 0.000057, 0.78, 0.81 (compared between with and without cold treatment; U test). ***P < 0.001. ns, P > 0.05. *n* = 83, 42, 45, 24, 51, 29, 23, 24.

Next, we asked how Sle is regulated. A hint came from our separate cytological screen for kinases or phosphatases that localize to the spindle area. In this screen, we live-imaged GFP-tagged proteins expressed from transgenes in metaphase I oocytes. Among them, GFP-Gwl showed a hollow spindle-like localization (Fig. 6 A). Gwl is a conserved serine/threonine kinase, which suppresses PP2A-B55, a major phosphatase counteracting Cdk1, in mitosis/meiosis (Castro and Lorca, 2018). Immunostaining with a newly generated Gwl antibody (Fig. S3, A and B) confirmed that the endogenous Gwl also surrounds the spindle (Fig. 7 A). Due to this and microtubule-independent localization described below, we refer to the structure or substance highlighted by Gwl as the Gwl spindle envelope in *Drosophila* oocytes. Previously, Axs was reported to surround the meiotic spindle in oocytes (Kramer and Hawley, 2003). Live imaging of Axs-GFP and mCherry-Gwl co-expressed in oocytes showed that they colocalize in metaphase I (Fig. 6, D and E).

**Figure 8.**
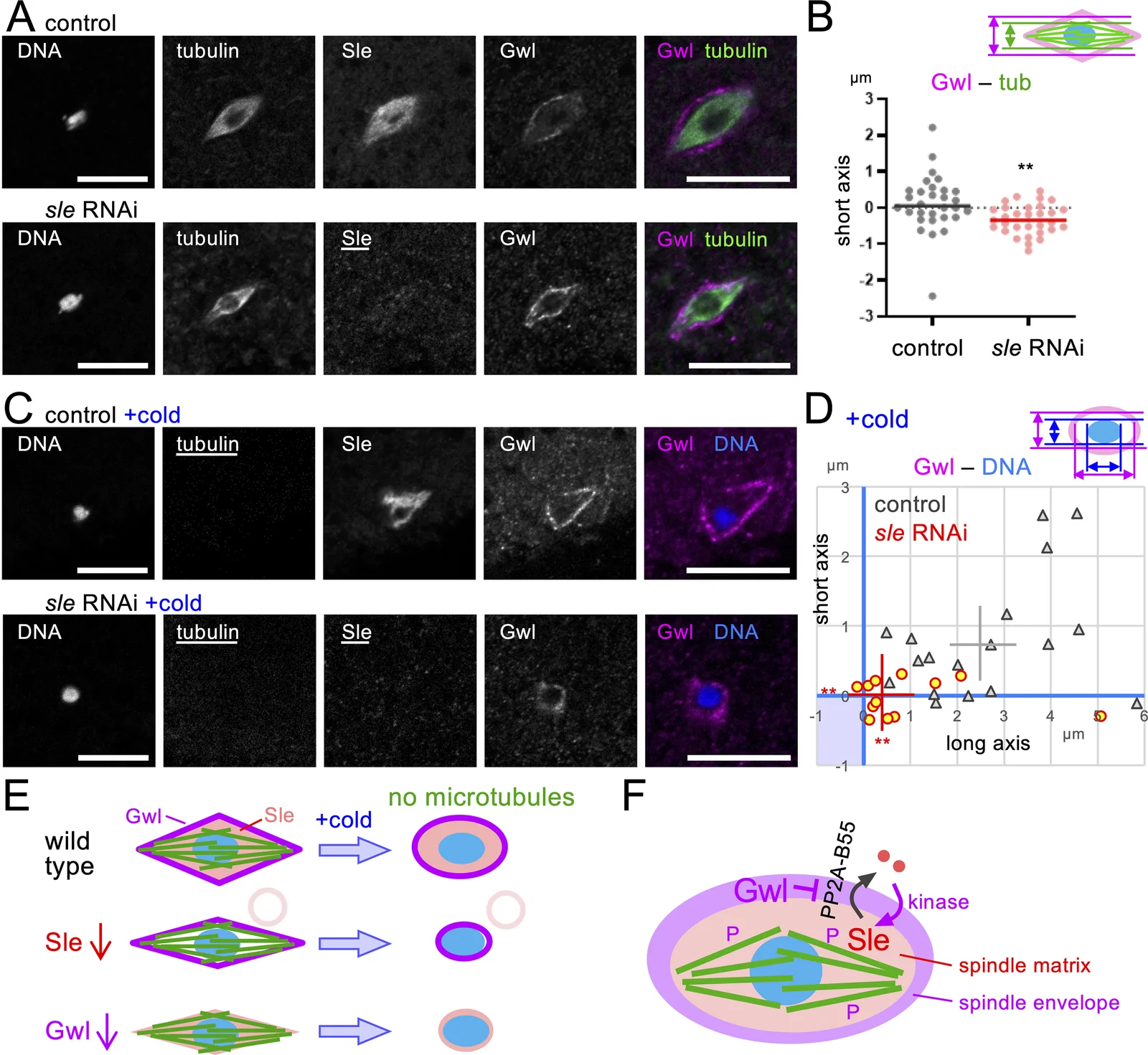
**Sle is an integral component of the spindle matrix, which determines the location of the Gwl spindle envelope. (A)** Localization of the endogenous Gwl in control and *sle*-silenced metaphase I oocytes. Oocytes were immunostained with antibodies against α-tubulin, Sle, and Gwl. Single focal planes are shown. The rightmost panels are merged images with a higher magnification than the others. Underlines indicate channels expected to have weak or diffused signals due to RNAi or depolymerization. Gwl collapsed onto spindle microtubules after *sle* silencing. Scale bars = 10 μm. **(B)** Width of the Gwl-surrounded area subtracted by the width of the spindle is plotted in control and *sle*-silenced (RNAi 1) oocytes. Underlines indicate channels expected to have weak or diffused signals due to RNAi or depolymerization. The red horizontal lines represent the median. *sle* silencing reduces the width of the Gwl-surrounded area compared with the spindle. P = 0.0016 (U test). **P < 0.01. *n* = 30, 31. **(C)** Localization of Gwl in control and *sle*-silenced (RNAi 1) metaphase I oocytes after microtubule depolymerization by cold treatment. Oocytes were immunostained with antibodies against α-tubulin, Sle, and Gwl. Single focal planes are shown. The rightmost panels are merged images with a higher magnification than the others. Underlines indicate channels expected to have weak or diffused signals due to RNAi or depolymerization. Gwl collapsed onto the chromosomes after *sle* silencing and microtubule depolymerization. Scale bars = 10 μm. **(D)** Long and short axes of the Gwl-surrounded area are subtracted by the long and short axes of the chromosome masses, respectively, in control and *sle*-silenced oocytes. Horizontal and vertical lines represent the medians. P = 0.0016, 0.0011 (long and short axes; U test). **P < 0.01. *n* = 18, 12 (control, *sle* RNAi). **(E)** Summary diagram of the effects of Sle or Gwl depletion on the Sle spindle matrix and the Gwl spindle envelope. **(F)** Gwl promotes the assembly of Sle into the spindle matrix by suppressing PP2A-B55.

No previous studies have simultaneously visualized both the spindle matrix and spindle envelope. To determine the relative localization, we immunostained oocytes expressing GFP-Gwl with antibodies against GFP, Sle, and tubulin, as well as wild-type oocytes with antibodies against Gwl, Sle, and tubulin. We found that Gwl tightly surrounded Sle, although Gwl and spindle microtubules had gaps between them in half of the oocytes (Fig. 6, A–C, Fig. 7 A, and Fig. 8 B).

To further test a relationship between the Sle spindle matrix and the Gwl spindle envelope, we depolymerized microtubules by cold treatment. The Sle spindle matrix remained as a mass around the chromosomes, as described previously (Fig. 1 C; Fig. 7 B). Gwl still tightly surrounded the Sle spindle matrix (Fig. 7 B). This suggests a close relationship between the locations of the Sle spindle matrix and the Gwl spindle envelope.

### Gwl promotes the assembly of the Sle spindle matrix in oocytes

These complementary localizations suggest a close relationship, but did not distinguish whether the Gwl spindle envelope defines the location of the Sle spindle matrix or *vice versa*. First, to determine the role of Gwl in the Sle spindle matrix, *gwl* was silenced by RNAi in oocytes (Fig. 7 and Fig. S3). The spindle often became wider or disorganized, and homologous centromeres were frequently mono-oriented (Fig. 7 C and Fig. S3, C–F). The broader defects in *gwl* silencing than *sle* silencing are likely because Gwl regulates multiple pathways in mitosis/meiosis (Castro and Lorca, 2018). Interestingly, Sle localization appeared to mirror more closely to the spindle microtubules (Fig. 7 C), rather than the wider localization often seen without *gwl* silencing (Fig. 7 A). This was confirmed by a quantitative comparison between the widths of Sle and the spindle (Fig. 7 I). After microtubule depolymerization by cold treatment, Sle collapsed onto the chromosomes and only occupied a small region around the chromosomes (Fig. 7 D). Quantitative analysis confirmed that *gwl* silencing dramatically reduced the area occupied by Sle and the total Sle signal intensity around the chromosomes compared with the control (Fig. 7, J and K). A western blot showed that *gwl* silencing did not alter the amount of Sle in ovaries (Fig. S3 B). In conclusion, Gwl kinase is not required for Sle localization to spindle microtubules, but is important for the full formation of the Sle spindle matrix.

The major downstream pathway of Gwl is to suppress the protein phosphatase PP2A-B55 (Tws) via phosphorylation of Endos in various animals including *Drosophila* (Rangone et al., 2011; Wang et al., 2013; García-Blanco et al., 2019). If Sle is regulated by this pathway, the defects seen in *gwl* silencing are caused by hyperactivation of PP2A-B55, and therefore can be rescued by downregulation of PP2A-B55. To test this possibility, *PP2A-B55* was cosilenced with *gwl* by expressing two shRNAs in oocytes. We found that cosilencing of *PP2A-B55* restored normal Sle localization in *gwl*-silenced oocytes both with and without cold treatment (Fig. 7, E, F, and I–K). This demonstrates that Gwl kinase promotes the assembly/stability of the Sle spindle matrix through suppression of PP2A-B55 in oocytes.

### Sle is an integral component of the spindle matrix, which determines the localization of the Gwl spindle envelope in oocytes

Next, to determine the role of Sle in the Gwl spindle envelope, *sle* was silenced in oocytes by RNAi (Fig. 8, A–D). Gwl still surrounded the spindle microtubules, but appeared to localize more tightly to the spindle in contrast to the no-RNAi control where gaps were often observed (Fig. 8 A). Gwl did not surround Sle bubbles (Fig. S3 G). Quantitative analysis confirmed that *sle* silencing significantly reduced the width of the Gwl-surrounded area compared with the spindle width (Fig. 8 B). When microtubules were depolymerized by cold treatment, Gwl collapsed onto the chromosomes and often appeared less organized in *sle*-silenced oocytes (Fig. 8 C) in contrast to the control where Gwl surrounded a larger area highlighted by Sle. Quantitative analysis confirmed that Gwl localized much closer to the chromosomes (Fig. 8 D).

This demonstrates that the Sle spindle matrix, spindle microtubules, and the chromosomes determine the location of the Gwl spindle envelope, whichever is the furthest out. Crucially, in the absence of microtubules, Sle depletion dramatically decreased the area surrounded by Gwl (which is normally occupied by the Sle spindle matrix). This suggests that Sle is important for the integrity of the spindle matrix, rather than simply associating with it without a structural role.

## Discussion

Historically, the study of the spindle structure and function centers around microtubules. Microtubule-independent structures, such as the spindle matrix and the spindle envelope, have been often overlooked (Kline-Smith and Walczak, 2004; Ferreira and Maiato, 2021; Valdez et al., 2023). In this study, we identify Sle as an integral component of the spindle matrix, and the Gwl kinase as a spindle envelope protein in metaphase I oocytes of *Drosophila*. We further showed that the Sle spindle matrix determines the location of the Gwl spindle envelope, and in turn, Gwl promotes assembly of the Sle spindle matrix. Sle is important for the spindle architecture but dispensable for bi-orientation of homologous centromeres in metaphase I oocytes. However, Sle becomes crucial for bi-orienting homologous centromeres when kinetochore–microtubule interaction is suboptimal.

Sle localizes not only to the spindle, but also to a wider area surrounding it. Furthermore, even after microtubules are depolymerized by cold treatment, Sle still occupies a sizable area around the chromosomes in oocytes. The Sle spindle matrix can be maintained independently of microtubules. At the same time, it interacts with spindle microtubules, as it shrinks on microtubule depolymerization. Live imaging in oocytes revealed that Sle is selectively stabilized or actively recruited to the spindle matrix, rather than simply failing to diffuse from the nucleus after NEBD. FRAP analysis also uncovered the dynamic nature of Sle in the spindle matrix in metaphase I. Exchanges of Sle take place mainly within the spindle matrix but also between the spindle matrix and the ooplasm. The composition of the spindle matrix in oocytes differs from mitosis. Sle is a common component for both, while Chro and Mgtor appear specific to the mitotic spindle matrix. Functional studies of mitotic spindle matrix proteins in oocytes are required to clarify involvement of these proteins in oocytes.

We also found that Gwl kinase localizes like the spindle envelope in oocyte meiosis. It colocalizes with Axs, a transmembrane protein previously reported to localize to a structure surrounding the spindle in oocytes (Kramer and Hawley, 2003). The spindle envelope and the spindle matrix have not been studied simultaneously in the same cell before. Our study shows that the Sle spindle matrix and the Gwl spindle envelope are interdependent (Fig. 8, E and F). Gwl kinase promotes assembly of Sle into the spindle matrix through suppressing PP2A-B55. Conversely, the location of the Gwl spindle envelope is determined by the Sle spindle matrix. The Gwl spindle envelope is located on the surface of the Sle spindle matrix. When Sle was depleted, the Gwl spindle envelope collapsed onto the spindle, and further onto the chromosomes upon microtubule depolymerization. Therefore, localization of the Gwl spindle envelope is determined first by the Sle spindle matrix; if it is missing, by the spindle microtubules; if both are missing, by the chromosomes.

Another important implication can be drawn from the observation that Sle depletion reduced the area surrounded by the Gwl spindle envelope (normally occupied by the Sle spindle matrix). This indicates that Sle is important for integrity of the spindle matrix in oocytes, rather than simply associating with the spindle matrix formed by other proteins. In other words, Sle is an integral component of the spindle matrix. Sle is a large protein mostly made of intrinsically disordered regions, and does not appear to be conserved beyond insects at the primary sequence level. FRAP analysis revealed that Sle exchanges predominantly within the spindle matrix but also slowly with the ooplasm. This might suggest that Sle assembles into the spindle matrix by forming viscous liquid-like condensates. Identification of Sle as an integral component is a crucial step for the study of the spindle matrix. Our study paves a way for functional evaluation, biophysical study, and *in vitro* reconstitution of the spindle matrix.

Gwl kinase is dispensable for Sle localization to spindle microtubules, but crucial for formation of the Sle spindle matrix. The surface of the Sle spindle matrix has a high concentration of Gwl kinase that promotes the assembly of the spindle matrix. As the surface is the place where assembly and disassembly of the spindle matrix likely take place, this Gwl localization on the surface may help the spindle matrix to assemble efficiently and resist disassembly or prevent diffusion (Fig. 8 F). A first step to test this hypothesis would be to clarify whether Gwl localization is important for these functions. We showed that Gwl kinase promotes assembly of the spindle matrix by suppressing the activity of protein phosphatase PP2A-B55. Therefore, the Sle spindle matrix assembly/disassembly is regulated by a balance between activities of an as yet unidentified kinase and this phosphatase. Cdk1 may be a prime candidate for this kinase, as PP2A-B55 is generally considered to be a major phosphatase to counteract Cdk1 (Castro and Lorca, 2018). Identifying the critical substrate, phospho-sites, and the kinase would be a crucial step to advance our limited understanding of the spindle matrix assembly.

Our study showed that Sle is important for the normal architecture of the metaphase I spindle in oocytes. After depleting Sle, despite structural defects, the spindle can still bi-orient homologous centromeres in meiotic metaphase I. Strikingly, when kinetochore–microtubule interaction is compromised, Sle becomes crucial for centromere bi-orientation. Due to the lack of centrosomes and the large volume of oocytes, dynamics of the spindle and the chromosomes depend on each other. This mutual dependency potentially makes them vulnerable to an internal or external disturbance. The microtubule-independent spindle matrix may act as a buffer to prevent them from spiraling out of control. Considering it as a safeguard, rather than as an essential structure, may be a more fruitful way to understand the spindle matrix. Further studies are required to clarify the precise role of the spindle matrix.

The spindle matrix and the spindle envelope have been known for decades, but a comprehensive understanding has eluded researchers. This study presents a new integrated framework to understand these two microtubule-independent structures as one functional unit in oocytes. Oocytes have an exceptionally large volume and lack a spatial landmark, the centrosome. The spindle matrix and the spindle envelope could help to generate a local environment favorable for spindle stability and function. Our study revealed their roles and functional relationship in *Drosophila* oocytes, and provides a conceptual and molecular basis for future studies.

## Materials and methods

### *Drosophila* techniques and genetics

Standard fly techniques were used throughout (Ashburner et al., 2011). All fly lines were maintained at 18°C on maize-based food and transferred to new vials every 4 wk. Working stocks were maintained at 25°C and transferred to new vials or bottles every 2 wk. All crosses were carried at 25°C, with the number of females doubling the number of males. For dissection, progeny were collected on the 10th day after crossing, and females were matured for 3 days (dissected on the 13th day after crossing) with half the number of males and additional dried yeast.

To depolymerize microtubules in oocytes, cold treatment was carried out as described previously (Romé and Ohkura, 2018). Matured flies were left in tubes at 4°C in a fridge or in a metal block on ice for 4 h before ovaries were dissected in cold methanol.

To drive RNAi, *y w; P{matα4-GAL4-VP16}V37* females were crossed with *y w; UASp-shRNA* males or *w*^*1118*^ males as a control. *sle* RNAi 1 was used to silence *sle* unless otherwise stated. To express GFP-tagged protein with Rcc1-mCherry for live imaging, *y w; P{matα4-GAL4-VP16}V37 {matα4-Rcc1-mCherry}::VK27* females (Costa and Ohkura, 2019) were crossed with *y w; GFP-XX* males, except FRAP analysis where *P{nos-GAL4-VP16}MVD1* females were crossed with *matα4-GFP-Sle {matα4-Rcc1-mCherry}*::*VK27* males.

The following fly stocks were obtained from Bloomington *Drosophila* Stock Center: *y w; P{matα4-GAL4-VP16}V37* (RRID:BDSC_7063), *w*^*1118*^*; P{w[+mC]=GAL4::VP16-nanos.UTR}CG6325[MVD1]/TM3, Sb* (RRID:BDSC_4937), *y sc v sev; P{y*^*+*^*v*^*+*^*=TRiP.HMS01578}attP2* (*tws*/*PP2A-B55* RNAi; RRID:BDSC_36689), and *y sc v sev; P{y*^*+*^*v*^*+*^*=TRiP.HMS00766}attP2* (*sle* RNAi B; RRID:BDSC_32969).

### Molecular methods

Standard molecular techniques (Sambrook, 1989) were used. The following recombinant DNA techniques were used for plasmid construction according to the manufacturers’ instructions: Gibson assembly (HiFi DNA Assembly; New England Biolabs), Gateway technology using LR Clonase II (Invitrogen), BP Clonase II (Invitrogen), Sanger sequencing (carried out by Genewiz), PCR using PrimeSTAR (Takara Bio) or Taq polymerase (Roche), and ligation using restriction enzymes (New England Biolabs) and T4 DNA ligase (New England Biolabs). Oligonucleotides were synthesized by IDT.

Proteins were analyzed by SDS-PAGE with a molecular weight marker (PageRuler 10–180 kDa; Thermo Fisher) and stained with InstantBlue Coomassie Protein Stain (Abcam). For western blotting, ovaries from 3-day-old mature females were dissected in methanol and rehydrated in PBS, homogenized, and incubated at 95°C for 3 min in the SDS sample buffer with 5% β-mercaptoethanol. A sample equivalent to one ovary was loaded to each lane for SDS-PAGE. Proteins were blotted onto nitrocellulose membranes (Protran; Whatman) and stained with Pierce Reversible Protein Stain Kit (Thermo Fisher). The membrane was blocked in PBS containing 0.05% Tween-20 and 3% skimmed milk (Marvel), and incubated with a primary antibody overnight, and a secondary antibody for >1 h. The membrane was washed multiple times in PBS+0.05% Tween-20 for a total of 30–60 min after each antibody incubation. A rat antibody against MBP-Sle-1 (this study; 1/250), a guinea pig antibody against GST-Gwl-dM1 (this study; 1/250), or a mouse antibody against α-tubulin (DM1A; T9026; Sigma-Aldrich; 1/250) was used as a primary antibody. Secondary antibodies conjugated with a fluorescent dye (IRDye800CW, IRDye680LT; LI-COR) were used at 1/20,000. After being rinsed in PBS, the signals were detected by Odyssey CLx (LI-COR) at a resolution of 168 μm. The total signal intensity of a band above the background was measured by the method described in image analysis.

### Generation of GFP- or mCherry-tagged lines

To generate plasmids expressing GFP- or mCherry-tagged proteins in flies, the coding sequence amplified from a template by PCR using PrimeSTAR (Takara Bio) was cloned between NotI and AscI sites of pENTR-D-Topo (Invitrogen) using the Gibson assembly. The templates used in this study were LD35132 (*gwl*), SD06626 (*Chro*), and genomic DNA of *w*^*1118*^ (*sle*). For *sle*, the start codon of RB was used. For *Mgtor*, an entry plasmid containing the full-length coding sequence was reconstructed by the Gibson assembly of three PCR-amplified fragments from SD15969 encoding an N-terminal part, a middle part from genomic DNA of *w*^*1118*^, and SD01380 encoding a C-terminal part. For *Axs*, the sequence from the start codon to the last coding codon was PCR-amplified from genomic DNA of *w*^*1118*^ using primers containing attB1 and attB2 and recombined into pDONR221 (Invitrogen) using BP Clonase (Invitrogen). After the full-coding sequence was reconstructed, this Gateway entry plasmid was recombined with a destination vector using LR Clonase II (Invitrogen). The destination vectors used in this study are pPWG and φPGW (Costa and Ohkura, 2019) containing UASp, and φMaChW and φMaGW (Costa and Ohkura, 2019) containing maternal *α4-tubulin at 67A* (*matα4*) promoter. φMaChW was generated by inserting PCR-amplified maternal *α4-tubulin at 67A* (*matα4*) promoter and mCherry into φMaGW digested with StuI and AgeI. The resulting plasmids were microinjected into the fly line (RRID:BDSC_9750; *PBac{y*^*+*^*-attP-3B}VK33*) or (RRID:BDSC_9744; *PBac{y*^*+*^*-attP-3B}VK27* for *matα4-*mCherry-Gwl) for φC31 integrase-mediated transgenesis or into *w*^*1118*^ for P-element transposase-mediated transgenesis, carried out by BestGene Inc. Resulting transgenic flies carrying *matα4-GFP-Sle*, *UASp-GFP-Gwl, UASp-GFP-Chro*, *UASp-Axs-GFP*, *matα4-mCherry-Gwl*, and *matα4-GFP-Mgtor* were used in this study.

### Generation of RNAi lines and double-RNAi screening

Transgenic RNAi fly lines were generated based on the RNAi vector pGP11 as previously described (Wan et al., 2025). Plasmids expressing a single shRNA under UASp were generated by inserting a pair of complementary oligonucleotides (Table S1) with cohesive ends between the EcoRI and NheI sites of pGP11 using T4 DNA ligase (NEB). To generate plasmids expressing two shRNAs against *sle* and a second gene, the region between EcoRI and NheI was amplified by PCR from a plasmid expressing shRNA against the second gene using PrimeSTAR (Takara) with flanking overlapping sequences for the Gibson assembly. The PCR product was then inserted into the SpeI site of a plasmid expressing a single shRNA against *sle* (RNAi 1) by the Gibson assembly. After the sequence was confirmed, the resulting plasmid was microinjected into the fly line (RRID:BDSC_8622; P{CaryP}attP2) using φC31 integrase-mediated transgenesis, carried out by BestGene Inc. The same procedure was used to construct the plasmid expressing two shRNAs against *gwl* and *tws*.

In the first round of double-RNAi screening, a small number of oocytes were subjected to FISH followed by immunostaining (immunoFISH) using dodecasatellite as a probe. When the frequency of mono-orientation was not significantly different (P > 0.05) by Fisher’s exact test between single RNAi of a gene and double RNAi with *sle*, it was not followed up further. For genes that showed a significant difference in the first round, more oocytes were examined in subsequent rounds by immunoFISH. Results were pooled together for Fisher’s exact test.

### Generation of antibodies

Two antibodies against Sle were generated as below. Expression plasmids encoding MBP-Sle-1 and GST-Sle-2 were introduced to *Escherichia coli BL21(DE3)/pLysS*. Sle-1 and Sle-2 encode 33–536 aa and 862–1,201 aa of Sle A isoform, respectively (Fig. S1 A). The bacteria were cultured overnight in LB with antibiotics at 37°C. It was diluted 100 times in the final volume of 200 ml LB with the antibiotics. Bacteria were collected by centrifugation and were resuspended in 3 ml lysis buffer (50 mM Tris-Cl, pH 8, 1 mM EDTA, 100 mM NaCl). It was then frozen and thawed until it became viscous. After the final concentration of 250 μg/ml lysozyme (Sigma-Aldrich) was added, it was incubated on ice for 10 min and at 37°C for another 10 min. Final 2 mM MgCl_2_ and 10 μg/ml DNase I (Sigma-Aldrich) were added, and the lysate was incubated at 37°C until viscosity disappeared. After 5 ml PBS was added, it was then centrifuged at 4°C for 10 min. The supernatant was run through a column containing 500 μl of glutathione Sepharose 4B resin (Cytiva) or 500 μl of amylose resin (New England Biolabs). Proteins were eluted five times with 10-min incubation of 500 μl of PBS containing 10 mM glutathione or 10 mM maltose. The eluted protein was analyzed by SDS-PAGE, and the concentration was estimated in comparison with BSA at known concentration. Two rats were immunized by Eurogentec using the classical 87-day program (50–100 μg of the antigen each injection), and antisera were used. The antiserum against MBP-Sle(33–536) from the final bleed of Rat 1 was used unless stated otherwise.

An antibody against Gwl was generated as described below. GST-Gwl lacking 224–640 residues (dM1) was expressed, and bacteria were lysed as described above for Sle proteins. After centrifugation following DNase I treatment and the addition of PBS, the pellet which contains most of the Gwl protein was then run on SDS-PAGE. The position of GST-Gwl-dM1 was identified on the gel by a difference in refraction of light. A slice containing the band was cut out and grinded. The protein was eluted by diffusion in 12 ml of 0.2 M NaHCO_3_+0.02% SDS at 37°C overnight. The elute was dialyzed in PBS, concentrated using Amicon Ultra-4 (Merck Millipore), and analyzed by SDS-PAGE with BSA for quantification. Two guinea pigs were immunized by Eurogentec using the classical 87-day program (50–100 μg of the antigen each injection). The antiserum against GST-Gwl-dM1 from the final bleed of guinea pig 6 was used.

An antibody against Ncd was prepared as described below. MBP-Ncd was expressed and purified as described above for GST-Gwl-dM1. This was used as the antigen to immunize rats as described above for the Sle antibodies. The final bleed from Rat0 was affinity-purified using the GST-tagged N-terminal nonmotor region of Ncd (1–349 aa) as previously described (Clohisey et al., 2014; Smith and Fisher, 1984). GST-Ncd(1–349) was expressed in *BL21(DE3)/pLysS*, and a crude extract containing ∼50 μg of GST-Ncd(1–349) was run on an SDS gel and transferred onto a nitrocellulose membrane. The membrane was stained with 1% Ponceau S in 1% acetic acid, and a slice of the membrane corresponding to GST-Ncd(1–349) was excised. After the membrane was washed and blocked in PBS containing 0.05% Tween-20 and 3% skimmed milk (Marvel), it was incubated with 100 μl of the final antiserum in 1 ml of blocking solution overnight at 4°C. After extensive washes in PBS+0.05% Tween-20, the antibody was eluted three times by 30-s incubation each with 400 μl elution buffer (50 mM glycine-HCl, pH 2.3, 0.5 M NaCl, 0.5% Tween-20, 100 μg/ml BSA, 0.1% NaN_3_). All of the eluates were combined and immediately neutralized by adding Na_2_HPO_4_ to a final concentration of 50 mM.

### Antibodies

The primary antibodies and concentrations used for immunostaining and western blot are rat anti-MBP-Sle1 (this study; 1/250), rat anti-GST-Sle-2 (this study; 1/250), guinea pig anti-MBP-Sle1 (this study; 1/250), rat anti-MBP-Ncd (this study; affinity-purified 1/10), guinea pig anti-GST-Gwl-dM1 (this study; 1/250), mouse anti-α-tubulin (DM1A; T9026; Sigma-Aldrich; RRID:AB_477593; 1/250), rabbit anti-GFP (A11122; Thermo Fisher Scientific; RRID:AB_221569; 1/125), mouse anti-Chro (Developmental Studies Hybridoma Bank [DSHB] 12H9; RRID:AB_2721936; 1/5), and mouse anti-Mgtor (DSHB 12H10; RRID:AB_2721935; 1/5), rabbit anti-CP190 (Rb188; 1/500; Whitfield et al., 1995).

The secondary antibodies used for immunostaining of oocytes and S2 cells were all from Jackson ImmunoResearch: anti-mouse IgG conjugated with Alexa Fluor 488 (715-546-151; RRID:AB_2340850; 1/100), anti-rabbit IgG conjugated with Alexa Fluor 488 (A-11034; 1/100), anti-rat IgG conjugated with Cy3 (712-166-153; RRID:AB_2340669; 1/500), anti-rabbit IgG conjugated with Cy3 (711-166-152; RRID:AB_2313568; 1/500), anti-guinea pig IgG conjugated with Cy3 (706-166-148; RRID:AB_2340461; 1/500), anti-rabbit IgG conjugated with Alexa Fluor 647 (711-606-152; RRID:2340625; 1/250), and anti-rat IgG conjugated with Alexa Fluor 647 (712-606-153; RRID:AB_2340696; 1/250). To detect GFP, a secondary antibody conjugated with Alexa Fluor 488 was always used. For western blot, secondary antibodies conjugated with a fluorescent dye (IRDye800CW) were used at 1/20,000 (RRID AB_268782, RRID AB_1850025, RRID AB_2814905, RRID AB_1850024; LI-COR).

### Cytological analysis of oocytes

After 3 days of maturation, ∼25 mature flies were quickly dissected and fixed in 100% methanol. The dissected ovaries were sonicated with 25–32% amplitude and three 1-s pulses using a VCX 750 SONICS sonicator to remove their chorion and vitelline membrane. Dechorionated oocytes were incubated in a mixture of half-volume methanol and half-volume PBS for 10 min, followed by incubation in 400 μl PBS for an additional 10 min. They were then blocked for 30 min in PBST-FBS blocking solution (PBS containing 0.1% Triton X-100 and 10% FBS). Oocytes were incubated in PBST (PBS containing 0.1% Triton X-100) with primary antibodies for 4 h overnight, washed in PBST three times for 10 min each, and then incubated in PBST with fluorophore-conjugated secondary antibodies and 0.5 μg/ml of 4′,6-diamidino-2-phenylindole (DAPI) in the dark for 2 h. They were then washed in PBST four times for 10 min each before being mounted in a mounting medium (85% glycerol, 2.5% propyl gallate). Prepared slides were imaged using a Plan-Apochromat objective lens (63×, 1.4NA) on AxioObserver 7 (Zeiss) attached to an LSM 900 (Zeiss) laser scanning confocal microscope controlled by ZEN (Zeiss) under Immersol 518F oil (Zeiss). Around 10–20 Z slices spanning the entire spindle were imaged with 0.5-μm interval at Zoom 2.0 (pixel size 0.10 μm), pinhole 53 μm, averaging 4× (repeat per line), master gain 500 V, digital offset 0, and digital gain 1.0. Typically, about 30 spindles were imaged from each batch and at least three batches were examined.

ImmunoFISH using centromere 3 probes was carried out as described previously (Repton et al., 2022). Ovaries were dissected in methanol, and oocytes were dechorionated as described for immunostaining. Oocytes were postfixed for 2 min with 8% PFA (15714; Electron Microscopy Sciences) in PBS. An oligonucleotide (5′-CCC​GTA​CTC​GGT-3′)_4_ was used as a probe for dodecasatellite near centromere 3. 100 pmol of the oligonucleotide was then end-labeled with 2 nmol Alexa 546–conjugated dUTP (Invitrogen) and 16 nmol of dTTP (Promega) using 30 units of terminal deoxynucleotidyl transferase (Promega) in 20 μl of transferase buffer at 37°C for one h. After incubating at 70°C for 10 min, free nucleotides were removed using a G25 Mini Quick spin column (Roche). The labeled probe was denatured at 91°C and incubated with ovaries in hybridization buffer (0.1 g/ml dextran sulfate, 50% formamide, 3x SSC) at 30°C overnight. After two washes in washing buffer (50% formamide, 3x SSC, 0.1% Triton X-100) at 30°C, oocytes were immunostained as described previously. Typically, about 30 spindles were imaged from each batch and at least three batches were examined except the first round of the double-RNAi screen. When two signals of homologous centromere 3 were fused together or located in the same half of the chromosome mass, it was classified as mono-orientation. When two signals were located in different halves of the chromosome mass, it was classified as bi-orientation.

For live-imaging oocytes, mature flies were dissected individually in oil (Halocarbon oil, 700) on day 2 or 3 of maturation. Stage 13 and 14 oocytes were attached onto a coverslip with their dorsal side facing down. The stage of oocytes was estimated based on dorsal appendages (Costa and Ohkura, 2019). Oocytes were live-imaged using a Plan-Apochromat objective lens (63×/1.4 NA) with 518F Immersol oil (Zeiss) on an AxioObserver Z.1 (Zeiss) microscope attached to a spinning disk confocal head (CSU-X1; Yokogawa) controlled by Volocity (Quorum). Around 10–20 Z slides were imaged with 0.8-μm interval (pixel size 0.1 μm), low light, Gain 0, Offset 0, and 0.25-s exposure. For live imaging of Axs-GFP and mCherry-Gwl, newly eclosed female flies were matured at 18°C for 6 days and at 25°C for 1–2 days, and 5- to 10-s exposure was used for imaging, to circumvent poor ovary development and weak signal.

All images are presented after maximum intensity projection, unless otherwise stated. For contrast changes, pixel intensities were changed only proportionally, except live images and western blot images where the pixel intensities were linearly changed between the maximum and minimal intensity values. All images were analyzed using ImageJ and edited using ImageJ or Omero. All scale bars are 10 μm, unless otherwise stated.

### FRAP analysis

For FRAP, oocytes were prepared as live imaging but imaged using a Plan-Apochromat objective lens (63×, 1.4NA) on Axio Observer 7 (Zeiss) attached to an LSM 900 (Zeiss) laser scanning confocal microscope controlled by ZEN (Zeiss) under Immersol 518F oil (Zeiss). Around 8–12 Z slices spanning the entire spindle were imaged every 5 s (for half spindle matrix bleach) or every 10 s (for whole spindle matrix bleach) with the following settings: 0.75-μm Z-interval, 256 × 256 pixels, pixel size 0.10 μm, pinhole 50 μm, no averaging, master gain 500 V, digital offset 0, and digital gain 1.0. For photobleaching, a rectangular box was drawn to cover half of the spindle matrix containing one pole (half matrix bleaching), or to cover the whole spindle matrix (whole matrix bleaching). This selected region on the middle Z-plane was bleached 3–10 times at 100% of the 488-nm laser power.

FRAP data were analyzed as follows. Before quantification, images were maximum intensity–projected onto XY planes, and a drift of the spindle over time was corrected using StackReg if required. For half spindle matrix bleach, two equal-sized rectangular boxes were drawn to include the bleached and the unbleached areas of the Sle spindle matrix. Two other rectangular boxes were drawn to include the whole spindle matrix and the background area. For whole spindle matrix bleach, two boxes including the whole matrix and background were drawn. The mean intensities within these boxes were measured. Firstly, for each oocyte, each intensity was divided by the background intensity at same time point to compensate for photobleaching by image acquisition. These compensated intensities were then divided by the prebleach values in each box, and then normalized using pre- and postbleach values of the bleached area. The average and standard errors of these normalized intensities were calculated from all oocytes and used to draw the graphs. For the lower left graph in Fig. 2 D, the average normalized intensity of the bleached area was subtracted by the average normalized intensity of the unbleached area at each time point, and further normalized using prebleach and postbleach values. For the lower right graph of Fig. 2 D, the average normalized intensities were further normalized using the average prebleach and postbleach normalized intensities in the whole spindle area.

To make a kymograph, the image at each time point was maximum intensity–projected first onto XY planes and then onto the spindle long axis. This one-dimensional image (on the x axis) was aligned with time (on the y axis). The “fire” lookup table was used to show intensity values after linear brightness adjustment.

### Fertility test and cytological analysis of syncytial embryos

To test female fertility, five adult females aged 1–4 days were placed with five sibling males in each vial containing fly food. After 6 days at 25°C, larval activity was checked, and after 12 days, the number of adult progeny from each vial was counted.

For immunostaining of early embryos, eggs were collected for 60 min on 3% agar plates containing 4% sucrose and food coloring, and further aged for 90 min at 25°C. They were dechorionated in 2.5% sodium hypochlorite, and fixed in bilayers of heptane and methanol containing 5 mM EGTA. Eggs without vitelline membranes were collected from the bottom of the methanol layer, and kept in fresh methanol overnight at −20°C. After gradually rehydrated in 80%, 60%, 40%, 20%, and 0% methanol in PBS, eggs were immunostained and observed as described for oocytes. Only one batch of egg collection was stained.

### Culture, transfection, and immunostaining of S2 cells

S2 cells were cultured in Schneider’s *Drosophila* medium (Gibco) with 10% FBS and 1% antibiotics (A5955; Sigma-Aldrich) at 27°C. Cells were split when they covered the bottom of T25 flasks containing a total volume of 10 ml (1/10 weekly).

To express GFP-tagged Sle, Chro, and Mgtor in S2 cells under the metallothionein promoter, expression plasmids were generated by recombining the entry plasmids described above with the destination vector pMT-GW (Goshima et al., 2007) using LR Clonase (Invitrogen). Each transfection mixture consisted of 300 μl of serum-free Schneider’s media, 600 ng of DNA (pMT-GFP-protein), and 1.8 μl of X-tremeGENE HP (Sigma-Aldrich) incubated at room temperature (∼21°C) for 25 min. The transfection mixture was then added to 10^6^ cells in each well of a 6-well plate. 500 μM of CuSO_4_ was added for induction, followed by adding 1 ml of Schneider’s media with FBS. Cells were fixed and immunostained after 24 h as described below.

1 ml of mixed cells (∼6 × 10^5^ cells) was added to the center of a concanavalin A–treated coverslip in a well, and 1 ml of warm media was added on the edges. After 2 h, cells were fixed with 3 ml cold methanol/PFA [90% methanol, 9% of 32% PFA (15714; Electron Microscopy Sciences), 1% of 0.5 M sodium bicarbonate (NaHCO_3_)] on dry ice. After 15 min, plates were placed at room temperature (∼21°C) for 15 min, then rinsed twice with PBS, and kept in PBS. For immunostaining, cells were permeabilized with PBST and blocked with a PBST-FBS blocking solution for 30 min. Cells were then incubated in the blocking solution with primary antibodies for 1 h, washed with PBST for 15 min, and incubated in a blocking solution with secondary antibodies, including 0.4 μg/ml DAPI. Typically, 10 or more mitotic cells were examined for each culture and two or more cultures were examined.

### Image analysis

In experiments with cold treatment, oocytes with residual spindle microtubules were excluded from analysis.

In Fig. 1 B and Fig. 6 C, signal intensities along the lines were measured using ImageJ plot profile, after the maximum intensity projection of the 3D image was generated. The intensities were normalized against the maximum intensity value of each channel and plotted against the positions on the line.

In Fig. 1 D, Fig. 7 K, and Fig. S2 E, to quantify the total signal intensity of an object above the background, two regions were drawn on an image after the maximum intensity projection as follows: a smaller region (S) closely surrounding the largest object from all the channels and a larger region (L) surrounding the smaller region. The same regions were used to measure the signals in all channels. The mean intensities and the sizes of the two regions were measured by ImageJ. The mean intensity of the background (B) was estimated by [(mean intensity L)x(area L)-(mean intensity S)x(area S)]/[(area L)-(area S)]. The total intensity of the object above the background was estimated by [(mean intensity S)-(mean intensity B)] × (area S). This value was divided by the mean intensity of the background for normalization. For S2 cells, the whole cell was used as L, except that if the cytoplasm has aggregates, they were excluded from L.

In Fig. 1 E, the lengths of the long and short axes of the spindle were measured after the maximum intensity projection by drawing two lines that pass in the middle of the DNA mass and span the longest and shortest lengths of the spindle, respectively. Spindles perpendicular to the focal planes were excluded from the analysis.

In Fig. 2 B and Fig. S2 A, intensities of GFP-Sle and Rcc1-mCherry over time were measured as follows. After the maximum intensity projection of the 3D images was generated, the average signal intensities within four squares of 9 × 9 pixels (0.9 × 0.9 μm) were measured using ImageJ and plotted over time. In Fig. S2, imaging was briefly interrupted to readjust the focus and/or stage during an interval of capturing. The intensities before the interruption were adjusted proportionally so that the intensities of the cytoplasmic background (box 1) were the same before and after the brief interruption.

Image analysis in Fig. 2, C and D, is separately described in the FRAP analysis section. In Fig. 4 A, the intensity of the Sle bands on the western blot above the background was measured by drawing a box of the same size around each band, and a smaller box in the background. The mean intensity within the box containing each band was subtracted by the mean intensity of the smaller box. The band intensities were indicated as values relative to the control.

In Fig. 4 C, the spindle morphology in *sle* RNAi oocytes was classified into the four following categories: smooth bipolar spindles with focused poles, bipolar spindles with thin microtubule bundles branching out from the sides, bipolar spindles with either split poles and/or many thin microtubule bundles branching out, and spindles with other abnormalities including nonbipolar spindles and multiple separate spindles. In Fig. S2 E, the spindle morphology in *gwl* RNAi oocytes was classified into the following categories: normal thin bipolar spindles, broader bipolar spindles, apolar spindles, and spindles with other abnormalities (including spindles with split poles or branched microtubule bundles, tripolar spindles, and multiple separate spindles).

In Fig. 5, A and C, orientation of homologous centromeres was operationally categorized as follows. When two signals of homologous centromere 3 were located closely together or in the same half of the chromosome mass along the long spindle axis, it was classified as mono-orientation. When two signals were located in different halves of the chromosome mass, it was classified as bi-orientation.

In Fig. 7 I, the width (the short axis) of the region occupied by Sle and the spindle microtubules was measured by drawing a line that passes the middle of the chromosome mass and spans the shortest length of the spindle after the maximum intensity projection. The same line was used to measure the widths in both channels. The spindle perpendicular to the focal planes was excluded from the analysis.

In Fig. 7 J, the length (the long axis) of the Sle region and the DNA mass was measured by drawing a line that passes in the middle of the DNA mass and spans the longest length of the Sle region after the maximum intensity projection. The same line was used to measure the lengths in both channels. Oocytes with an unclear Sle border were excluded from the analysis. In case multiple DNA masses were surrounded by separate Sle regions, the largest Sle region and the associated DNA mass were measured.

In Fig. 8 B, the width of the spindle and the region surrounded by the Gwl spindle envelope was measured after maximum intensity projection as follows. A line was drawn to pass the middle of the DNA mass and span the shortest width of the spindle. The inner width of the Gwl envelope and the outer width of the spindle were measured. The same line was used to measure the widths in different channels.

In Fig. 8 D, the length and width of the DNA mass and the region surrounded by the Gwl spindle envelope were measured after maximum intensity projection as follows. Two lines were drawn to pass the middle of the DNA mass and span the longest and shortest lengths of the Gwl-surrounded region, respectively. The same lines were used for measurement in different channels. The inner length/width of the Gwl envelope and the outer length/width of the DNA mass were measured. Oocytes with disorganized Gwl that does not surround the chromosomes were excluded from the measurement.

To statistically test differences between a pair of datasets, a two-tailed Mann–Whitney U test (https://www.statskingdom.com) was used for nonparametric data in Fig. 1 D; Fig. 7, I–K; Fig. 8, B and D; and Fig. S3 F, and Fisher’s exact test (https://www.medcalc.org/) was used for categorical data in Fig. 4, C and E; Fig. 5, A and C; Fig. S2 D; and Fig. S3, D and E. The frequencies of categorical data are shown with 95% confidence intervals (Wilson score intervals; https://www.statskingdom.com) in Fig. 4, C and E; Fig. 5, A and C; and Fig. S3, D and E.

### Online supplemental material

Fig. S1 shows Sle antibodies. Fig. S2 shows Sle localization and function in mitotic cells and oocytes. Fig. S3 shows Gwl antibody, function, and localization. Table S1 shows oligonucleotide sequences used for generating RNAi constructs.

## Supplementary Material

Review History

Table S1 shows oligonucleotide sequences used for generating RNAi constructs.

SourceData F4 is the source file for Fig. 4.

SourceData FS1 is the source file for Fig. S1.

SourceData FS3 is the source file for Fig. S3.

## Data Availability

Data supporting the findings of this study are available in the main text or the supplementary materials. Additional raw data are available from the corresponding author upon request.

## References

[bib1] Ashburner, M., K. Golic, and R.S. Hawley. 2011. *Drosophila*: A Laboratory Handbook. Cold Spring Harbor Laboratory Press, New York.

[bib2] Castro, A., and T. Lorca. 2018. Greatwall kinase at a glance. J. Cell Sci. 131:jcs222364. 10.1242/jcs.222364 30355803

[bib3] Civelekoglu-Scholey, G., L. Tao, I. Brust-Mascher, R. Wollman, and J.M. Scholey. 2010. Prometaphase spindle maintenance by an antagonistic motor-dependent force balance made robust by a disassembling lamin-B envelope. J. Cell Biol. 188:49–68. 10.1083/jcb.200908150 PMC2812851 20065089

[bib4] Clohisey, S.M.R., N.S. Dzhindzhev, and H. Ohkura. 2014. Kank is an EB1 interacting protein that localises to muscle-Tendon attachment sites in *Drosophila*. PLoS One. 9:e114770. 10.1371/journal.pone.0106112 PMC4159139 25203404

[bib5] Colombié, N., C.F. Cullen, A.L. Brittle, J.K. Jang, W.C. Earnshaw, M. Carmena, K. McKim, and H. Ohkura. 2008. Dual roles of incenp crucial to the assembly of the acentrosomal metaphase spindle in female meiosis. Development. 135:3239–3246. 10.1242/dev.022624 PMC2536564 18755775

[bib6] Costa, M.F.A., and H. Ohkura. 2019. The molecular architecture of the meiotic spindle is remodeled during metaphase arrest in oocytes. J. Cell Biol. 218:2854–2864. 10.1083/jcb.201902110 PMC6719438 31278080

[bib7] Dumont, J., and A. Desai. 2012. Acentrosomal spindle assembly and chromosome segregation during oocyte meiosis. Trends Cell Biol. 22:241–249. 10.1016/j.tcb.2012.02.007 PMC3348331 22480579

[bib8] Ferreira, L.T., and H. Maiato. 2021. Prometaphase. Semin. Cell Dev. Biol. 117:52–61. 10.1016/j.semcdb.2021.06.004 34127384

[bib9] Forer, A. 1965. Local reduction of spindle fiber birefringence in living *Nephrotoma suturalis* (Loew) spermatocytes induced by ultraviolet microbeam irradiation. J. Cell Biol. 25:95–117. 10.1083/jcb.25.1.95 PMC2106607 14342833

[bib10] García-Blanco, N., A. Vázquez-Bolado, and S. Moreno. 2019. Greatwall-endosulfine: A molecular switch that regulates PP2A/B55 protein phosphatase activity in dividing and quiescent cells. Int. J. Mol. Sci. 20:6228. 10.3390/ijms20246228 PMC6941129 31835586

[bib11] Głuszek, A.A., C.F. Cullen, W. Li, R.A. Battaglia, S.J. Radford, M.F. Costa, K.S. McKim, G. Goshima, and H. Ohkura. 2015. The microtubule catastrophe promoter Sentin delays stable kinetochore-microtubule attachment in oocytes. J. Cell Biol. 211:1113–1120. 10.1083/jcb.201507006 PMC4687879 26668329

[bib12] Goldman, R.D., and L.I. Rebhun. 1969. The structure and some properties of the isolated mitotic apparatus. J. Cell Sci. 4:179–209. 10.1242/jcs.4.1.179 4180667

[bib13] Goshima, G., R. Wollman, S.S. Goodwin, N. Zhang, J.M. Scholey, R.D. Vale, and N. Stuurman. 2007. Genes required for mitotic spindle assembly in *Drosophila* S2 cells. Science. 316:417–421. 10.1126/science.1141314 PMC2837481 17412918

[bib14] Huang, Y., T. Li, S.C. Ems-McClung, C.E. Walczak, C. Prigent, X. Zhu, X. Zhang, and Y. Zheng. 2018. Aurora A activation in mitosis promoted by BuGZ. J. Cell Biol. 217:107–116. 10.1083/jcb.201706103 PMC5748987 29074706

[bib15] Jiang, H., S. Wang, Y. Huang, X. He, H. Cui, X. Zhu, and Y. Zheng. 2015. Phase transition of spindle-associated protein regulate spindle Apparatus assembly. Cell. 163:108–122. 10.1016/j.cell.2015.08.010 PMC4607269 26388440

[bib16] Johansen, J., and K.M. Johansen. 2009. The spindle matrix through the cell cycle in *Drosophila*. Fly (Austin). 3:213–220. 10.4161/fly.3.3.9340 19690461

[bib17] Johansen, K.M., A. Forer, C. Yao, J. Girton, and J. Johansen. 2011. Do nuclear envelope and intranuclear proteins reorganize during mitosis to form an elastic, hydrogel-like spindle matrix? Chromosome Res. 19:345–365. 10.1007/s10577-011-9187-6 21274615

[bib18] Johansen, K.M., and J. Johansen. 2007. Cell and molecular biology of the spindle matrix. Int. Rev. Cytol. 263:155–206. 10.1016/S0074-7696(07)63004-6 17725967

[bib19] Kline-Smith, S.L., and C.E. Walczak. 2004. Mitotic spindle assembly and chromosome segregation: Refocusing on microtubule dynamics. Mol. Cell. 15:317–327. 10.1016/j.molcel.2004.07.012 15304213

[bib20] Kramer, J., and R.S. Hawley. 2003. The spindle-associated transmembrane protein Axs identifies a membranous structure ensheathing the meiotic spindle. Nat. Cell Biol. 5:261–263. 10.1038/ncb944 12646877

[bib21] Kyogoku, H., and T.S. Kitajima. 2023. The large cytoplasmic volume of oocyte. J. Reprod. Dev. 69:1–9. 10.1262/jrd.2022-101 PMC9939283 36436912

[bib22] Lee, W.L., and P. Wadsworth. 2009. New spindle morphogenesis model by Dynein, Nudel, and the spindle matrix. Cell Res. 19:529–531. 10.1038/cr.2009.49 PMC2848825 19421240

[bib23] Lince-Faria, M., S. Maffini, B. Orr, Y. Ding, C. Florindo, C.E. Sunkel, Á. Tavares, J. Johansen, K.M. Johansen, and H. Maiato. 2009. Spatiotemporal control of mitosis by the conserved spindle matrix protein megator. J. Cell Biol. 184:647–657. 10.1083/jcb.200811012 PMC2686406 19273613

[bib24] Ma, L., M.-Y. Tsai, S. Wang, B. Lu, R. Chen, J.R. Yates, X. Zhu, and Y. Zheng. 2009. Requirement for Nudel and dynein for assembly of the lamin B spindle matrix. Nat. Cell Biol. 11:247–256. 10.1038/ncb1832 PMC2699591 19198602

[bib25] Ohkura, H. 2015. Meiosis: An overview of key differences from mitosis. Cold Spring Harb. Perspect. Biol. 7:a015859. 10.1101/cshperspect.a015859 PMC4448623 25605710

[bib26] Orihara-Ono, M., E. Suzuki, M. Saito, Y. Yoda, T. Aigaki, and C. Hama. 2005. The slender lobes gene, identified by retarded mushroom body development, is required for proper nucleolar organization in *Drosophila*. Dev. Biol. 281:121–133. 10.1016/j.ydbio.2005.02.020 15848394

[bib27] Pickett-Heaps, J.D., A. Forer, and T. Spurck. 1997. Traction fibre: Toward a “tensegral” model of the spindle. Cell Motil. Cytoskeleton. 37:1–6. 10.1002/(SICI)1097-0169(1997)37:1<1::AID-CM1>3.0.CO;2-D 9142434

[bib28] Qi, H., U. Rath, Y. Ding, Y. Ji, M.J. Blacketer, J. Girton, J. Johansen, and K.M. Johansen. 2005. EAST interacts with megator and localizes to the putative spindle matrix during mitosis in *Drosophila*. J. Cell Biochem. 95:1284–1291. 10.1002/jcb.20495 15962301

[bib29] Qi, H., U. Rath, D. Wang, Y.-Z. Xu, Y. Ding, W. Zhang, M.J. Blacketer, M.R. Paddy, J. Girton, J. Johansen, and K.M. Johansen. 2004. Megator, an essential coiled-coil protein that localizes to the putative spindle matrix during mitosis in *Drosophila*. Mol. Biol. Cell. 15:4854–4865. 10.1091/mbc.E04 PMC524733 15356261

[bib30] Radford, S.J., T.L. Hoang, A.A. Głuszek, H. Ohkura, and K.S. McKim. 2015. Lateral and end-on kinetochore attachments are coordinated to achieve bi-orientation in *Drosophila* oocytes. PLoS Genet. 11:e1005605. 10.1371/journal.pgen.1005605 PMC4608789 26473960

[bib31] Rangone, H., E. Wegel, M.K. Gatt, E. Yeung, A. Flowers, J. Debski, M. Dadlez, V. Janssens, A.T.C. Carpenter, and D.M. Glover. 2011. Suppression of scant identifies endos as a substrate of greatwall kinase and a negative regulator of protein phosphatase 2a in mitosis. PLoS Genet. 7:e100225. 10.1371/journal.pgen.1002225 PMC3154957 21852956

[bib32] Rath, U., D. Wang, Y. Ding, Y.-Z. Xu, H. Qi, M.J. Blacketer, J. Girton, J. Johansen, and K.M. Johansen. 2004. Chromator, a novel and essential chromodomain protein interacts directly with the putative spindle matrix protein skeletor. J. Cell Biochem. 93:1033–1047. 10.1002/jcb.20243 15389869

[bib33] Repton, C., C. Fiona Cullen, M.F.A. Costa, C. Spanos, J. Rappsilber, and H. Ohkura. 2022. The phospho-docking protein 14-3-3 regulates microtubule-associated proteins in oocytes including the chromosomal passenger Borealin. PLoS Genet. 18:e1009995. 10.1371/journal.pgen.1009995 PMC9203013 35666772

[bib34] Romé, P., and H. Ohkura. 2018. Combining microscopy and biochemistry to study meiotic spindle assembly in *Drosophila* oocytes. Methods Cell Biol. 145:237–248. 10.1016/bs.mcb.2018.03.026 PMC6031290 29957206

[bib35] Sambrook, J. 1989. Molecular Cloning. Cold Spring Harbor Laboratory Press, New York.

[bib36] Schweizer, N., N. Pawar, M. Weiss, and H. Maiato. 2015. An organelle-exclusion envelope assists mitosis and underlies distinct molecular crowding in the spindle region. J. Cell Biol. 210:695–704. 10.1083/jcb.201506107 PMC4555823 26304726

[bib37] Schweizer, N., M. Weiss, and H. Maiato. 2014. The dynamic spindle matrix. Curr. Opin. Cell Biol. 28:1–7. 10.1016/j.ceb.2014.01.002 24491920

[bib38] Smith, D.E., and P.A. Fisher. 1984. Identification, developmental regulation, and response to heat shock of two antigenically related forms of a major nuclear envelope protein in *Drosophila* embryos: Application of an improved method for affinity purification of antibodies using polypeptides immobilized on nitrocellulose blots. J. Cell Biol. 99, 20-28. 10.1083/jcb.99.1.20 PMC2275611 6203917

[bib39] So, C., K.B. Seres, A.M. Steyer, E. Mönnich, D. Clift, A. Pejkovska, W. Möbius, and M. Schuh. 2019. A liquid-like spindle domain promotes acentrosomal spindle assembly in mammalian oocytes. Science. 364:eaat9557. 10.1126/science.aat9557 PMC6629549 31249032

[bib40] Tsai, M.-Y., S. Wang, J.M. Heidinger, D.K. Shumaker, S.A. Adam, R.D. Goldman, and Y. Zheng. 2006. A mitotic lamin b matrix induced by RanGTP required for spindle assembly. Science. 311:1887–1893. 10.1126/science.1122771 16543417

[bib41] Valdez, V.A., L. Neahring, S. Petry, and S. Dumont. 2023. Mechanisms underlying spindle assembly and robustness. Nat. Rev. Mol. Cell Biol. 24:523–542. 10.1038/s41580-023-00584-0 PMC10642710 36977834

[bib42] Walker, D.L., D. Wang, Y. Jin, U. Rath, Y. Wang, J. Johansen, and K.M. Johansen. 2000. Skeletor, a novel chromosomal protein that redistributes during mitosis provides evidence for the formation of a spindle matrix. J. Cell Biol. 151: 1401–1412. 10.1083/jcb.151.7.1401 PMC2150677 11134070

[bib43] Wan, X., G. Pavlova, C.F. Cullen, I. Dasuzhau, A. Ciszek, and H. Ohkura. 2025. Identification of locally activated spindle-associated proteins in oocytes uncovers a phosphatase-driven mechanism. J. Cell Sci. 138:jcs264161. 10.1242/jcs.264161 PMC12669969 41047934

[bib44] Wang, P., J.A. Galan, K. Normandin, É. Bonneil, G.R. Hickson, P.P. Roux, P. Thibault, and V. Archambault. 2013. Cell cycle regulation of Greatwall kinase nuclear localization facilitates mitotic progression. J. Cell Biol. 202:277–293. 10.1083/jcb.201211141 PMC3718974 23857770

[bib45] Whitfield, W.G., M.A. Chaplin, K. Oegema, H. Parry, and D.M. Glover. 1995. The 190 kDa centrosome-associated protein of *Drosophila* melanogaster contains four zinc finger motifs and binds to specific sites on polytene chromosomes. J. Cell Sci. 108:3377–3387. 10.1242/jcs.108.11.3377 8586650

[bib46] Woodruff, J.B. 2018. Assembly of mitotic structures through phase separation. J. Mol. Biol. 430:4762–4772. 10.1016/j.jmb.2018.04.041 29751016

[bib47] Yao, C., C. Wang, Y. Li, Y. Ding, U. Rath, S. Sengupta, J. Girton, K.M. Johansen, and J. Johansen. 2014. The spindle matrix protein, chromator, is a novel tubulin binding protein that can interact with both microtubules and free tubulin. PLoS One. 9:e103855. 10.1371/journal.pone.0103855 PMC4114980 25072297

[bib48] Zheng, Y. 2010. A membranous spindle matrix orchestrates cell division. Nat. Rev. Mol. Cell Biol. 11:529–535. 10.1038/nrm2919 PMC5474289 20520622

