# Supplementary material for: Slender lobes is a spindle matrix protein regulated by spindle envelope kinase Greatwall in oocytes
Source: J Cell Biol. 2026 Oct 26;226(1):e202606020. doi: 10.1083/jcb.202606020 (PMC13701656; doi:10.1083/jcb.202606020)
Supplement: Supplementary file 1 [file jcb_202606020_review_history.pdf]

Slender lobes is a spindle matrix protein regulated by spindle envelope kinase Greatwall in oocytes

Rayane Kaade, Emma Peat, Charlotte Repton, Julia Niziol, Carmela Mason, Li Ern Yap, Gera Pavlova, and Hiroyuki Ohkura

Corresponding Author(s): Hiroyuki Ohkura, University of Edinburgh

Review Timeline:

Submission Date:	2026-06-03
Editorial Decision:	2026-07-13
Revision Received:	2026-09-01
Editorial Decision:	2026-09-21
Revision Received:	2026-09-30

Monitoring Editor: Arshad Desai

Scientific Editor: Gabriele Stephan

Transaction Report:

(Note: With the exception of the correction of typographical or spelling errors that could be a source of ambiguity, letters and reports are not edited. The original formatting of letters and referee reports may not be reflected in this compilation.)

DOI: https://doi.org/10.1083/jcb.202606020

July 13, 2026

Re: JCB manuscript #202606020

Hiroyuki Ohkura
University of Edinburgh

Dear Prof. Ohkura,

Thank you for submitting your manuscript entitled "Slender lobes is a spindle matrix protein regulated by spindle envelope kinase Greatwall in oocytes". The manuscript was assessed by expert reviewers, whose comments are appended to this letter. Based on their feedback, we invite you to submit a revision addressing the reviewers comments.

You will see that all reviewers agree that your paper provides new insights towards understanding the spindle matrix and the spindle envelope. The reviewers provide constructive feedback we recommend that you address their comments. These include experimental extensions recommended by reviewers #1 and #2, such as those related the recruitment and liquid-like behavior of Sle, the effect of Sle and Gwl RNAi on chromosome orientation and greater clarity on localization and function of B55. Information on biological replicates and quantifications, as requested by reviewer #3, as well as all recommended textual revisions should also be included in the revised manuscript.

While you are revising your manuscript, please also attend to the following editorial points to help expedite the publication of your manuscript. Please direct any editorial questions to the journal office.

GENERAL GUIDELINES:

Text limits: Character count for an Article is < 40,000, not including spaces. Count includes title page, abstract, introduction, results, discussion, and acknowledgments. Count does not include materials and methods, figure legends, references, tables, or supplemental legends.

Figures: Articles may have up to 10 main text figures. Figures must be prepared according to the policies outlined in our Instructions to Authors, under Data Presentation, https://jcb.rupress.org/site/misc/ifora.xhtml. All figures in accepted manuscripts will be screened prior to publication.

IMPORTANT: It is JCB policy that if requested, original data images must be made available. Failure to provide original images upon request will result in unavoidable delays in publication. Please ensure that you have access to all original microscopy and blot data images before submitting your revision.

Supplemental information: There are strict limits on the allowable amount of supplemental data. Articles may have up to 5 supplemental figures. Up to 10 supplemental videos or flash animations are allowed. A summary of all supplemental material should appear at the end of the Materials and methods section.

Please note that JCB now requires authors to submit Source Data used to generate figures containing gels and Western blots with all revised manuscripts. This Source Data consists of fully uncropped and unprocessed images for each gel/blot displayed in the main and supplemental figures. For assays performed using capillary electrophoresis and/or immunoassay-based detection, authors should instead provide the electropherogram graph(s) for each experiment, plotting fluorescence/chemiluminescence intensity vs. molecular weight/size. Please be sure to provide one Source Data file for each figure gels, blots, and/or capillary electrophoresis assays along with your revised manuscript files. File names for Source Data figures should be alphanumeric without any spaces or special characters (i.e., SourceDataF#, where F# refers to the associated main figure number or SourceDataFS# for those associated with Supplementary figures). For traditional gels and blots, the lanes of the gels/blots should be labeled as they are in the associated figure, the place where cropping was applied should be marked (with a box), and molecular weight/size standards should be labeled wherever possible. For capillary electrophoresis assays, each trace in the graph should be color-coded and labeled to indicate which protein, gene, or sample is being measured (please try to avoid red/green combinations to accommodate our color-blind readers).

Source Data files will be made available to reviewers during evaluation of revised manuscripts and, if your paper is eventually published in JCB, the files will be directly linked to specific figures in the published article.

Source Data Figures should be provided as individual PDF files (one file per figure). Authors should endeavor to retain a minimum resolution of 300 dpi or pixels per inch. Please review our instructions for export from Photoshop, Illustrator, and PowerPoint here: https://rupress.org/jcb/pages/submission-guidelines#revised

The typical timeframe for revisions is three to four months. If you anticipate any difficulties in meeting this aforementioned

revision time limit, please contact us and we can work with you to find an appropriate time frame for resubmission. Please note that papers are generally considered through only one revision cycle, so any revised manuscript will likely be either accepted or rejected.

When submitting the revision, please include a cover letter addressing the reviewers' comments point by point. Please also highlight all changes in the text of the manuscript.

We hope that the comments below will prove constructive as your work progresses. We would be happy to discuss them further once you've had a chance to consider the points raised in this letter.

Thank you for this interesting contribution to Journal of Cell Biology. You can contact us at the journal office with any questions at cellbio@rockefeller.edu.

Sincerely,

Arshad Desai
Monitoring Editor
Journal of Cell Biology

Gabriele Stephan
Scientific Editor
Journal of Cell Biology

Reviewer #1:

The spindle matrix is a structure surrounding spindle microtubules whose composition and function remain debated. Here, the authors identify Slender lobes (Sle), a nucleolar protein lacking well-characterized domains, as a novel component of the spindle matrix. Through rigorous experiments combining genetics, immunofluorescence, and live imaging, they demonstrate that Sle localizes to the spindle matrix in both oocytes and S2 cells, distinguishing it from previously identified spindle matrix components such as Megator and Chromator, which are restricted to S2 cells and absent from oocytes. They further show that Sle depletion impairs spindle pole focusing but affects homologous chromosome orientation only when kinetochore-microtubule interactions are compromised. Finally, the authors decipher the interactions between the spindle envelope, enriched in Greatwall kinase, and the spindle matrix containing Sle. I really liked this manuscript which presents in a very clear and rigorous manner the identification of a novel ubiquitous spindle matrix component and identifies both its mode of regulation and its function. I only have very minor comments on the work.

1/ If possible, could the authors perform FRAP of GFP-Sle, to see active recruitment of the spindle associated pool? Eventually, could they show more dynamic GFP-Sle movies when it becomes enriched in the spindle? It seems indeed that Sle forms liquid like droplets in the spindle area (Fig 1A and 1C). Is it just an impression? Such a liquid-like behavior is reminiscent of the liquid-like domain observed at the poles of the spindle of mouse oocytes (So Science 2019). Is Sle conserved in mamals?

2/ We suggest showing immunofluorescence stainings of Sle, Mtor, and Chro in inverted LUT in Figures 3A-F, and providing quantifications comparing spindle versus cytoplasmic enrichment for these three proteins in mitosis and oocytes, where applicable.

3/ since Sle is also a component of the spindle matrix in S2 cells, does it have a different function than its role in oocyte meiosis. Is the Greatwall/Sle interaction also conserved and important in mitosis?

Reviewer #2:

This paper nicely defines two structures that in the past have been associated with a variety of structures and functions - the spindle matrix and spindle envelope. This work arose from the identification of Slender lobes (Sle) as a component of the spindle matrix in oocytes. The authors also identified a component of the spindle envelope, Gwl kinase. It is striking that the matrix maintains some of its structure in the absence of microtubules. The strength of this paper is a nice characterization of Sle and Gwl localization and dynamics during spindle assembly. The authors also show a loss of Sle causes abnormalities in spindle organization, although surprisingly, little effect on chromosome alignment except in combination with other knockdowns. They also define evidence of interactions between the spindle, spindle matrix and spindle envelope. As part of this work, the authors generated several useful reagents such as antibodies, new RNAi and GFP lines. For all these conclusions, the data is strongly supportive. This is also a well written paper and I have only minor criticisms.

1) The effect of the sle single RNAi on the orientation of homologous chromosomes is weak and no other proteins are identified as part of the spindle matrix. Can the authors integrate Axs into this story? Does it have a similar localization pattern to gwl?. Can the authors test if achiasmate chromosome segregation is affected by Sle RNAi. Does gwl RNAi have an effect on chromosome orientation?

2) Pg 7, lines 3-11: There is important information missing about the phenotype of Sle RNAi. What is the fertility of RNAi females? Is there a function after meiosis I such as in anaphase, meiosis II, fertilization or embryonic development? The authors should comment on the differences compared to the published null mutants? Does sle have a function outside of germline?

3) pg 8, line 5: I think the authors too quickly dismiss the relationship between Ndc80 and cmet depleted oocytes. Both could have defects in making end-on attachments. Is it possible that lateral attachments are very sensitive to sle depletion? A minor note, the phenotype of HURP, Mklp2, kinesin-5 and Spc105R could be removed from the graph because they are too severe to observe an enhancement.

4) Pg 9, Line 11: How does gwl silencing effect Sle? One might expect the envelope to restrict the matrix, but instead the opposite is observed. Can the authors confirm this and speculate a little? Is it possible that the Gwl activity, but not the actual structure of the envelope is important to regulate sle? Or does Gl stabilize Sle, and the most stable mirrors the spindle mt localization.

5) The result with the gwl B55 double is nice. But Fig 7 is missing data on B55 RNAi. Can the authors add localization data for B55?

6) Can the authors speculate or even examine the effect of kinases known to be antagonized by B55. While Cdk1 could be hard to test, experiments with Cyclins A, B or B3 are possible.

7) Pg 11, line 16-17: Perhaps summarize the differences between oocytes and mitosis? Also discuss some limitations of this study- since the only data for some proteins is a lack of localization, but not functional data.

8) In the methods, or in Figure 5, the identity of the RNAi lines should be provided.

Reviewer #3:

The manuscript under review by Kaade et. al. focuses on the contributions of Sle (slender lobes) and Gwl (greatwall) kinase to Drosophila oocyte meiosis. The authors show that Sle is a component of a microtubule-independent "spindle matrix" while Gwl forms a "spindle envelope" that surrounds the matrix. Depletion of Gwl reduced the size of the Sle matrix, and depletion of Sle reduced the size of the spindle envelope, suggesting that the matrix determines the location of the envelope. Moreover, depletion of Sle and Gwl (separately) resulted in spindle defects, highlighting roles for these structures in oocytes. I think that this manuscript reports interesting findings that will be of interest to the cell division and meiosis fields, as these enigmatic matrix/envelope structures have been reported for many years but are still poorly understood. However, I have a number of comments (detailed below) that I think would improve the rigor and clarity of the manuscript prior to publication.

Specific points:
1. The authors state that "Gwl is required for the assembly of Sle into the spindle matrix" (pg. 4, line 26). However, following Gwl depletion, Sle is still spindle-localized (Figure 7C). It is true that in the absence of Gwl, Sle does not extend as far out past the tubulin intensity, so the matrix may be smaller. However, since Sle still forms some sort of structure (that persists after cold treatment), it seems like an overstatement, here and at other points in the manuscript, to imply that Gwl is required for the matrix.
2. Related to point #1, if Gwl is required for the assembly of Sle into the matrix, then shouldn't the phenotypes of Gwl depletion and Sle depletion be similar? The authors show that there are spindle defects in both cases, but in the case of Sle it seems to be disorganized spindles/split poles (Figure 4C), while Gwl depletion seems to cause mostly wider spindles (Figure S2C). These differences should be noted and potential causes for the phenotypic differences proposed (and incorporated into the model), to help reader comprehension.
3. Page 5, line 15: the authors state that Sle localization is broader than the spindle, and callout Figures 1A and B. However, these panels only show one image (and the resulting linescan), while the quantification is not shown until Figure 7G. I know it might be odd to put a callout to Figure 7 here, out of order, but I do think that the reader would be more convinced of the conclusion if these data were referenced at this point in the paper. Another possibility would be to add a graph/panel to Figure 1, showing the control data from Fig 7G (without the mutant data). Then, in the legend for 7G, you could put a note that the control data is repeated from Figure 1 as a comparison to the mutants.
4. Page 5, line 15: Related to this control data from Figure 7G, the text states that the localization of Sle is broader "mostly up to 1um". This wording is a bit confusing. Do you mean that most of the data points are between 0-1um? Maybe it could be simpler to just report the average and put a callout to Figure 7G.
5. Figure 2 only shows a single timelapse video to support the results discussed on page 6, lines 3-14. Additional replicates

should be imaged (and quantification of those movies shown) to support the claims. Since this imaging may be technically challenging, the number of additional movies does not have to be high. However, it is not sufficient to only show one movie.
6. Page 6, line 8-9: The authors claim that Sle begins accumulating around the chromosomes ~10 minutes after nuclear envelope breakdown. However, the quantification of fluorescence intensity (shown in Figure 2B) does not include an ROI around the chromosomes, which would be required to support this claim.
7. Page 6, line 21: It would be helpful to adjust the wording to "Sle colocalized with these two proteins in metaphase..." for clarity, since Sle does not appear to co-localize with these proteins in interphase.
8. Figure 3 only shows a single representative image for each of the conditions. The authors should state how many oocytes they imaged for each condition, to make this data more convincing (e.g. adding something like "each image is representative of data obtained from at least xx oocytes" to the figure legend). This is especially important when trying to show that a protein doesn't localize somewhere - what if the antibody staining just didn't work on the one slide that the image came from? As a related point, I think that it is important that the data throughout the paper is from multiple biological replicates. I am assuming that this is true, but to give the reader confidence that the conclusions are sound, the authors should note in the materials and methods how many biological replicates they typically do for each type of technique (immunofluorescence, live imaging, etc). If the number varies between figures (and therefore cannot be generalized by technique), then this information could be added to each figure legend instead.
9. Figure 4: I found the labeling of the 4 RNAi lines to be a bit odd: why is one "B" and the others are "1, 2, 3"? Is it because the publicly available line had a different notation? This is not a big deal, but to avoid this minor confusion on the part of the reader, it might be nice to add a sentence to the figure legend to explain.
10. Figure 4: It is clear from the quantification that the number of normal bipolar spindles is decreased following Sle depletion. However, why are there so many abnormal spindles in the control? It would help to acknowledge this somewhere in the paper, and speculate on the reason, to avoid reader confusion.
11. The finding that Sle co-depletion with kinetochore components (Ndc80 and Cenp-E) increases mono-orientation is interesting. However, it looks like the same was not true for Spc105. Could this be because mono-orientation was already so high with Spc105 depletion alone that it was difficult to see an enhancement of the phenotype? It might be worth briefly speculating on this, for clarity.
12. Page 9, line 5: the claim that spindles were often wider following gwl depletion should be supported with quantification. I saw that spindle morphology was quantified in Figure S2C, but since that quantification is by eye, it is unclear what the cutoff between "normal" and "wide" was. Since the authors did measure spindle widths (because they used those values for the Sle-tubulin quantification in Figure 7G), it should be straightforward to include a graph of spindle widths in the various mutants. Beyond supporting the initial statement about spindles getting wider following gwl depletion, it would also be interesting to see if the wide spindle phenotype was rescued by gwl B55 codepletion.
13. Figure 6: only a single image is shown and quantified. The authors should provide some indication of how many oocytes they looked at and how many looked like the representative image (especially since on page 8, line 30, they state that "Gwl and spindle microtubules often had gaps between them" - how can you say "often" if you only quantify one image?).
14. Figure 8E: This is minor, but is there a reason why Gwl is drawn as a diamond shape in the Sle depletion + cold condition? The image in Figure 8C looks like Gwl just collapses into a circle around the chromosomes under this condition, so I was not sure what the diamond was meant to represent.

Typos:
- Page 5, line 11: "relatively" should be "relative"
- Page 5, line 21: "Ncd were" should be "Ncd was"
- Page 5, line 33: "previously located" should be "was previously located"
- Page 7, line 23: "the spindle architecture" should be "spindle architecture"
- Page 11, line 19: "fails" should be "failing"

JCB manuscript #202606020

"Slender lobes is a spindle matrix protein regulated by spindle envelope kinase Greatwall in oocytes" Kaade et al.

Dear Editors,

We are very pleased to have received amazingly positive comments from all three reviewers together with constructive comments, and to be given an opportunity to further improve the manuscript. Following your advice, we have incorporated all of the reviewers' comments. Firstly, we have added several new experimental data (listed below) as suggested by reviewers. Secondly, we have included further quantification data (listed below) and information such as sample numbers. Thirdly we have modified text and figures to improve clarity. Point-by-point response to each of the reviewers' comments can be found in the next page.

These changes and addition of new experimental results have greatly improved the manuscript, which we believe is now suitable for publication in *Journal of Cell Biology*.

Hiro

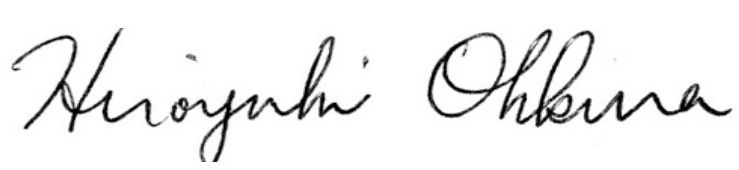

Hiro Ohkura
Professor of Cell Biology
Centre for Cell Biology
The University of Edinburgh
Edinburgh EH9 3BF, UK
+44-131-650-7094
h.ohkura@ed.ac.uk

[Experimental data added to the revised version]

- Two additional live movies of GFP-Sle from NEBD and quantification (new Fig.S2A)
- FRAP analysis of GFP-Sle in oocytes, showing dynamic property (new Fig.2C-E)
- Defects of *sle* RNAi in fertility and early embryonic divisions (new Fig.S2B-D)
- Live-imaging of Axs-GFP and mCherry-Gwl co-expressed in oocytes, showing their colocalisation to the spindle envelope (Fig.6D,E)
- Frequency of centromere mono-orientation in *gwl* RNAi (new Fig.S3D)
- Sle localisation in *PP2A-B55* RNAi oocytes with and without cold treatment, and quantification (Fig.7G-K)

[New quantitative analysis added to the revised version]

- Sle, Chro, Mgtor intensities from a number of mitotic cells and oocytes (new Fig.S2E)
- The spindle widths and morphologies in *gwl*, *B55* and *gwl B55* RNAi oocytes, showing the rescue of the *gwl* RNAi phenotype by *PP2A-B55* RNAi (new Fig.S3E,F).

Point-by-point response to the reviewers' comments

Reviewer #1:

"I really liked this manuscript which presents in a very clear and rigorous manner the identification of a novel ubiquitous spindle matrix component and identifies both its mode of regulation and its function. I only have very minor comments on the work."

We are pleased to hear very positive comments and constructive suggestions which we have fully incorporated into the revised manuscript as below, including addition of new experimental data.

"1/ If possible, could the authors perform FRAP of GFP-Sle... It seems indeed that Sle forms liquid like droplets in the spindle area (Fig 1A and 1C). Is it just an impression? ... Is Sle conserved in mamals?"

As suggested, we have carried out FRAP analysis of GFP-Sle in metaphase I oocytes. It revealed a dynamic property of Sle. It exchanges predominantly within the spindle matrix, but also slowly with the ooplasm. This suggests that the Sle within the spindle matrix shows a dynamic, (viscous) liquid-like property, rather than forms a solid structure. It also suggests that Sle is constantly but slowly recruited to and released from the spindle matrix.

Our results are consistent with (but not exclusive to) liquid-liquid phase separation, which is an attractive hypothesis but is often controversial due to lack of definitive methods to prove. Therefore, we are cautious about making such a claim at this stage. FRAP data is now presented in Fig.2C-E, and this hypothesis is now included in Discussion.

From the primary sequence, we could not find homologues beyond insects. However, as Sle is nearly entirely made of intrinsically disordered regions, the sequence-based search may not reveal homologues even if any. This information is now included in Discussion.

"2/ We suggest showing immunofluorescence stainings of Sle, Mtor, and Chro in inverted LUT in Figures 3A-F, and providing quantifications comparing spindle versus cytoplasmic enrichment for these three proteins in mitosis and oocytes, where applicable."

We have inverted LUT in Fig.3A-F and included quantification of the Sle, Mgtor and Chro intensities from multiple cells and oocytes in Fig.S2E, as suggested. This has improved the clarity and rigor of the data.

"3/ since Sle is also a component of the spindle matrix in S2 cells, does it have a different function than its role in oocyte meiosis. Is the Greatwall/Sle interaction also conserved and important in mitosis?"

Mitotic roles and regulation of Sle is a potentially fertile area of study. As suggested, we carried out preliminary investigation in S2 cells, which has not yet revealed clear spindle function or Sle-Gwl interaction (detailed below). We need in-depth study to make a firm conclusion, and consider it beyond the scope of this manuscript. As requested by another reviewer, we also examined syncytial mitosis in embryos from *sle* RNAi mothers. Syncytial divisions showed abnormalities, suggesting roles of Sle beyond female meiosis. Images and quantification showing abnormalities in early embryonic divisions have been added as Fig.S2C,D.

sle RNAi in S2 cells did not appear to give obvious spindle defects, although subtle defects may be hidden by a high background of spindle abnormalities in untreated S2 cells. Immunostaining showed that Gwl weakly localise to the spindle rather than surrounding the Sle spindle matrix, and this localisation did not change in *sle* RNAi. Conversely, Sle localisation pattern did not change in *gwl* RNAi, although the signal might be weaker. These differences between S2 cells and oocytes might be because the spindle matrix in mitosis contains additional components, or mitotic cells are small and have centrosomes. Further studies are required.

Reviewer #2:

"This paper nicely defines two structures... The strength of this paper is a nice characterization of Sle and Gwl localization and dynamics during spindle assembly.... For all these conclusions, the data is strongly supportive. This is also a well written paper and I have only minor criticisms."

We are delighted to hear very positive comments and constructive suggestions which we have fully incorporated into the revised manuscript as detailed below, including addition of new experimental data.

"1) ...Can the authors integrate Axs into this story? Does it have a similar localization pattern to gwl?. Can the authors test if achiasmate chromosome segregation is affected by Sle RNAi. Does gwl RNAi have an effect on chromosome orientation?"

To define Axs localisation relative to Gwl, we first tried to generate an Axs antibody without success. Then we made transgenic lines simultaneously expressing mCherry-Gwl and Axs-GFP, but initially suffered from poor ovary development and weak signals. Finally we succeeded in seeing co-localisation of Axs and Gwl to the spindle envelope (n=6). The data is now included in Fig.6D,E.

We carried out FISH of centromere 3 in *gwl* RNAi oocytes, which showed an elevated frequency (28%) of centromere mono-orientation. This result is now included in Fig.S3F. We could not test achiasmatic chromosome segregation, as the *sle* RNAi line is nearly infertile.

"2) Pg 7, lines 3-11: ... What is the fertility of RNAi females? Is there a function after meiosis I such as in anaphase, meiosis II, fertilization or embryonic development? The authors should comment on the differences compared to the published null mutants? Does sle have a function outside of germline?"

sle RNAi females are nearly infertile, consistent with a previous report showing that a null mutant is viable but infertile. The null mutant has underdeveloped ovaries, but we found that our RNAi line lays eggs which fail to hatch. Immunostaining of these eggs showed frequent abnormalities in syncytial mitotic divisions. Fertility test and abnormalities in early embryonic divisions have been added with images and quantification as Fig.S2B-D.

"3) pg 8, line 5: I think the authors too quickly dismiss the relationship between Ndc80 and cmet depleted oocytes. Both could have defects in making end-on attachments. Is it possible that lateral attachments are very sensitive to sle depletion? A minor note, the phenotype of HURP, Mklp2, kinesin-5 and Spc105R could be removed from the graph because they are too severe to observe an enhancement."

We agree that we should not exclude a possibility that a particular mode of kinetochore-microtubule interaction may be sensitive to *sle* RNAi. We have modified the text accordingly. Despite a suggestion, we retain the data of the genes with the most severe defects in the graph to show that the defect of *cmet sle* double RNAi nearly reaches the maximum level.

"4) Pg 9, Line 11: How does gwl silencing effect Sle? One might expect the envelope to restrict the matrix, but instead the opposite is observed. Can the authors confirm this and speculate a little? Is it possible that the Gwl activity, but not the actual structure of the envelope is important to regulate sle? Or does Gl stabilize Sle, and the most stable mirrors the spindle mt localization."

It is possible that the spindle envelope may restrict the spindle matrix to a confined space to prevent it from diffusing out into the large ooplasm and shrinking the spindle matrix. It is also possible that Gwl activity, not the structure of the spindle envelope, is important. There are many interesting possibilities and questions to how Gwl regulates Sle, which we continue to investigate beyond this paper. Discussion was modified to mention some of the possibilities and give more emphasis on limitation of our understanding.

"5) The result with the gwl B55 double is nice. But Fig 7 is missing data on B55 RNAi. Can the authors add localization data for B55?"

We looked at the localisation of Sle in *B55* single RNAi oocytes with and without cold treatment. *B55* single RNAi did not significantly change the Sle localisation. This is probably because the B55 phosphatase activity is already suppressed by Gwl and further suppression has a minimal effect on Sle. These data have been added with images and graphs to Fig.7.

"6) Can the authors speculate or even examine the effect of kinases known to be antagonized by B55. While Cdk1 could be hard to test, experiments with Cyclins A, B or B3 are possible."

Cdk1 RNAi prevents the nuclear envelope breakdown. Then we tested an available *Cyclin B* RNAi line, but it did not appear to alter the spindle matrix with or without cold treatment. Further studies are required to clarify whether Cdk1 is the kinase antagonised by B55 for the spindle matrix. This possibility is now suggested in Discussion.

"7) Pg 11, line 16-17: Perhaps summarize the differences between oocytes and mitosis? Also discuss some limitations of this study- since the only data for some proteins is a lack of localization, but not functional data."

We have added a summary statement of the difference between oocytes and mitosis, and a statement on a need for functional studies in Discussion.

"8) In the methods, or in Figure 5, the identity of the RNAi lines should be provided."

All RNAi lines in Figure 5 were generated for this study in our lab. Exact shRNA sequences used for generating each line are described in the supplementary table (Table S1), which was omitted from the PDF of the original manuscript.

Reviewer #3:

"I think that this manuscript reports interesting findings that will be of interest to the cell division and meiosis fields, as these enigmatic matrix/envelope structures have been reported for many years but are still poorly understood. However, I have a number of comments (detailed below) that I think would improve the rigor and clarity of the manuscript prior to publication."

We are pleased to hear very positive comments and constructive suggestions which we have fully incorporated into the revised manuscript as detailed below, including addition of experimental data and quantifications. It has greatly improved the rigor and clarity of the manuscript.

Specific points:

"1. ... it seems like an overstatement, here and at other points in the manuscript, to imply that Gwl is required for the matrix."

We fully agree that this is a careless statement. We have changed it to "important".

"2. ... shouldn't the phenotypes of Gwl depletion and Sle depletion be similar? ...These differences should be noted and potential causes for the phenotypic differences proposed (and incorporated into the model), to help reader comprehension."

During M phase, Gwl suppresses PP2A-B55 which is considered to be the main phosphatase counteracting Cdk1. Therefore, Gwl depletion is expected to have much wider impacts than a failure to assemble the Sle spindle matrix alone. This information has been added to Results.

"3. Page 5, line 15: the authors state that Sle localization is broader than the spindle, ... the quantification is not shown until Figure 7G.."

"4. Page 5, line 15: ...the text states that the localization of Sle is broader "mostly up to 1um". This wording is a bit confusing. ... Maybe it could be simpler to just report the average and put a callout to Figure 7G."

As suggested, we now state the median value (0.53 μm) with reference to Fig.7G.

"5. Figure 2 only shows a single timelapse video to support the results discussed on page 6, lines 3-14. Additional replicates should be imaged (and quantification of those movies shown) to support the claims... "

We have had three more timelapse videos, which also share the key features of the video shown in Fig.2. Two of them have sufficient quality for intensity quantification over time. The quantified data is now included in Fig.S2.

"6. Page 6, line 8-9: The authors claim that Sle begins accumulating around the chromosomes ~10 minutes after nuclear envelope breakdown. However, the quantification of fluorescence intensity (shown in Figure 2B) does not include an ROI around the chromosomes, which would be required to support this claim."

We did not include the spindle area in the measurement, because the position of the chromosomes significantly changes over time. Sle initially appeared as just small dots, which is indicated by an arrowhead in the Fig.2A. We quantified time between NEB and initiation of spindle assembly from the four videos (11.0±1.2 min; mean±SD) and have included this in the text.

"7. Page 6, line 21: It would be helpful to adjust the wording to "Sle colocalized with these two proteins in metaphase..." for clarity, since Sle does not appear to co-localize with these proteins in interphase."

We have added "in metaphase" to the text for clarity.

"8. Figure 3 only shows a single representative image for each of the conditions. The authors should state how many oocytes they imaged for each condition, ... the authors should note in the materials and methods how many biological replicates they typically do for each type of technique (immunofluorescence, live imaging, etc).... "

The numbers of samples we observed have been added to the figure legend. Furthermore, we have quantified the signal intensities and included the data as Fig.S2E. More generally, information on the sample numbers and biological replicates we typically examined have been added to the Materials and Methods. Three or more replicates were typically examined in oocytes

with some exceptions such as the first round of the double RNAi mini-screen in Fig.5 (25-30 oocytes from 20-30 flies were stained together). In S2 cells, two or more replicates were typically examined.

"9. Figure 4: I found the labeling of the 4 RNAi lines to be a bit odd: why is one "B" and the others are "1, 2, 3"? Is it because the publicly available line had a different notation?... "

RNAi B is from Bloomington Stock Center, while 1,2 and 3 were generated by us. As 1,2 and 3 used an expression vector different from B, we thought it is better to have distinct names. The information has been added to the legend.

"10. Figure 4: It is clear from the quantification that the number of normal bipolar spindles is decreased following Sle depletion. However, why are there so many abnormal spindles in the control? It would help to acknowledge this somewhere in the paper, and speculate on the reason, to avoid reader confusion."

We avoid saying "abnormal", as most of them are seen also in wild-type oocytes but the frequencies are higher in *sle* RNAi. Meiotic spindles are very dynamic. In live imaging, it is common to see a stray microtubule bundle transiently appears but is quickly absorbed back to the main spindle in wild type. We now state the dynamic nature of the meiotic spindle in the figure legend.

"11. ... Could this be because mono-orientation was already so high with Spc105 depletion alone that it was difficult to see an enhancement of the phenotype? It might be worth briefly speculating on this, for clarity."

Spc105R depletion showed one of the most severe phenotypes. As suggested, it may not be possible to enhance the phenotype even further. This speculation has been included in Results.

"12. Page 9, line 5: the claim that spindles were often wider following gwl depletion should be supported with quantification. ... it would also be interesting to see if the wide spindle phenotype was rescued by gwl B55 codepletion."

As suggested, we measured the spindle width from the *gwl* single RNAi and *gwl B55* double RNAi, which showed that co-depletion of B55 rescued the spindle width phenotype of *gwl* RNAi. The data is now included in Fig.S3 (previously Fig.S2).

"13. Figure 6: only a single image is shown and quantified. The authors should provide some indication of how many oocytes they looked at and how many looked like the representative image (especially since on page 8, line 30, they state that "Gwl and spindle microtubules often had gaps between them" - how can you say "often" if you only quantify one image?)."

Fig.8B in the original manuscript contains quantified data, which shows that 15 out of 30 oocytes have a gap between Gwl and the spindle. However, we agree that this is too late for readers, and the information is now included in page 8 with reference to Fig.8B.

"14. Figure 8E: This is minor, but is there a reason why Gwl is drawn as a diamond shape in the Sle depletion + cold condition? The image in Figure 8C looks like Gwl just collapses into a circle around the chromosomes under this condition, so I was not sure what the diamond was meant to represent."

In some cases, there appear to have small gaps between the Gwl envelope and the chromosomes (can be seen in Fig.8D). However, we agree that a diamond shape is confusing. We have modified the diagram in Fig.8E.

"Typos:
- Page 5, line 11: "relatively" should be "relative"
- Page 5, line 21: "Ncd were" should be "Ncd was"
- Page 5, line 33: "previously located" should be "was previously located"
- Page 7, line 23: "the spindle architecture" should be "spindle architecture"
- Page 11, line 19: "fails" should be "failing" "

Thank you for pointing out our errors. We have corrected them accordingly.

September 21, 2026

RE: JCB Manuscript #202606020R

Hiroyuki Ohkura
University of Edinburgh

Dear Prof. Ohkura:

Thank you for submitting your revised manuscript entitled "Slender lobes is a spindle matrix protein regulated by spindle envelope kinase Greatwall in oocytes". Your manuscript was re-reviewed by two of the original referees, who now support publication, so we would be happy to publish your paper in JCB pending final revisions necessary to meet our formatting guidelines (see details below).

To avoid unnecessary delays in the acceptance and publication of your paper, please read the following information carefully.

A. MANUSCRIPT ORGANIZATION AND FORMATTING:

Full guidelines are available on our Instructions for Authors page, http://jcb.rupress.org/submission-guidelines#revised.
Submission of a paper that does not conform to JCB guidelines will delay the acceptance of your manuscript.

1) Text limits: Character count for Articles is < 40,000, not including spaces. Count includes abstract, introduction, results, discussion, and acknowledgments. Count does not include title page, figure legends, materials and methods, references, tables, or supplemental legends.

2) Figures limits: Articles may have up to 10 main text figures.

3) Figure formatting: Scale bars must be present on all microscopy images, including inset magnifications. Molecular weight or nucleic acid size markers must be included on all gel electrophoresis. Aspect ratios of images may not be altered.

**4) Statistical analysis: Error bars on graphic representations of numerical data must be clearly described in the figure legend. The number of independent data points (n) represented in a graph must be indicated in the legend.
**Statistical methods should be explained in full in the materials and methods. For figures presenting pooled data the statistical measure should be defined in the figure legends. Please also be sure to indicate the statistical tests used in each of your experiments (either in the figure legend itself or in a separate methods section) as well as the parameters of the test (for example, if you ran a t-test, please indicate if it was one- or two-sided, etc.). Also, if you used parametric tests, please indicate if the data distribution was tested for normality (and if so, how). If not, you must state something to the effect that "Data distribution was assumed to be normal but this was not formally tested."

5) Abstract and title: The abstract should be no longer than 160 words and should communicate the significance of the paper for a general audience. The title should be less than 100 characters including spaces. Make the title concise but accessible to a general readership.

**6) Materials and methods: Should be comprehensive and not simply reference a previous publication for details on how an experiment was performed. Please provide full descriptions in the text for readers who may not have access to referenced manuscripts. For example, in the "Drosophila techniques and genetics" paragraph of your methods section, you state: "To depolymerise microtubules in oocytes, cold treatment was carried out as described previously (Romé & Ohkura, 2018)." You must remove this and write out the full methodology. The same goes for all other places in the methods where you refer to methods "...as previously described."

7) All antibodies, cell lines, animals, and tools used in the manuscript should be described in full, including accession numbers for materials available in a public repository such as the Resource Identification Portal. Please be sure to provide the sequences for all of your primers/oligos and RNAi constructs in the materials and methods. You must also indicate in the methods the source, species, and catalog numbers (where appropriate) for all of your antibodies. Please also indicate the acquisition and quantification methods for immunoblotting/western blots.

8) Microscope image acquisition: The following information must be provided about the acquisition and processing of images:
a. Make and model of microscope
b. Type, magnification, and numerical aperture of the objective lenses
c. Temperature
d. Imaging medium
e. Fluorochromes

f. Camera make and model
g. Acquisition software
h. Any software used for image processing subsequent to data acquisition. Please include details and types of operations involved (e.g., type of deconvolution, 3D reconstitutions, surface or volume rendering, gamma adjustments, etc.).

9) References: There is no limit to the number of references cited in a manuscript. References should be cited parenthetically in the text by author and year of publication. Abbreviate the names of journals according to PubMed.

10) Supplemental materials: There are strict limits on the allowable amount of supplemental data. Articles may have up to 5 supplemental figures. Please also note that tables, like figures, should be provided as individual, editable files. A summary of all supplemental material should appear at the end of the Materials and methods section.

11) eTOC summary: A ~40-50-word summary that describes the context and significance of the findings for a general readership should be included on the title page. The statement should be written in the present tense and refer to the work in the third person.

12) Conflict of interest statement: JCB requires inclusion of a statement in the acknowledgements regarding competing financial interests. If no competing financial interests exist, please include the following statement: "The authors declare no competing financial interests." If competing interests are declared, please follow your statement of these competing interests with the following statement: "The authors declare no further competing financial interests."

**13) ORCID IDs: ORCID IDs are unique identifiers allowing researchers to create a record of their various scholarly contributions in a single place. Please note that ORCID IDs are now *required* for all authors. At resubmission of your final files, please be sure to provide your ORCID ID and those of all co-authors.

14) A separate author contribution section following the Acknowledgments. All authors should be mentioned and designated by their full names. We encourage use of the CRediT nomenclature.

Please note that JCB now requires authors to submit Source Data used to generate figures containing gels and Western blots with all revised manuscripts. This Source Data consists of fully uncropped and unprocessed images for each gel/blot displayed in the main and supplemental figures. For assays performed using capillary electrophoresis and/or immunoassay-based detection, authors should instead provide the electropherogram graph(s) for each experiment, plotting fluorescence/chemiluminescence intensity vs. molecular weight/size. Please be sure to provide one Source Data file for each figure gels, blots, and/or capillary electrophoresis assays along with your revised manuscript files. File names for Source Data figures should be alphanumeric without any spaces or special characters (i.e., SourceDataF#, where F# refers to the associated main figure number or SourceDataFS# for those associated with Supplementary figures). For traditional gels and blots, the lanes of the gels/blots should be labeled as they are in the associated figure, the place where cropping was applied should be marked (with a box), and molecular weight/size standards should be labeled wherever possible. For capillary electrophoresis assays, each trace in the graph should be color-coded and labeled to indicate which protein, gene, or sample is being measured (please try to avoid red/green combinations to accommodate our color-blind readers).

Source Data files will be made available to reviewers during evaluation of revised manuscripts and, if your paper is eventually published in JCB, the files will be directly linked to specific figures in the published article.

Source Data Figures should be provided as individual PDF files (one file per figure). Authors should endeavor to retain a minimum resolution of 300 dpi or pixels per inch. Please review our instructions for export from Photoshop, Illustrator, and PowerPoint here: https://rupress.org/jcb/pages/submission-guidelines#revised

Journal of Cell Biology now requires a data availability statement for all research article submissions. These statements will be published in the article directly above the Acknowledgments. The statement should address all data underlying the research presented in the manuscript. Please visit the JCB instructions for authors for guidelines and examples of statements at (https://rupress.org/jcb/pages/editorial-policies#data-availability-statement).

B. FINAL FILES:

Please upload the following materials to our online submission system. These items are required prior to acceptance. If you have any questions, contact JCB's Managing Editor, Lindsey Hollander (lhollander@rockefeller.edu).

-- An editable version of the final text (.DOC or .DOCX) is needed for copyediting (no PDFs).

-- High-resolution figure and MP4 video files: See our detailed guidelines for preparing your production-ready images, https://jcb.rupress.org/fig-vid-guidelines.

-- Cover images: If you have any striking images related to this story, we would be happy to consider them for inclusion on the journal cover. Submitted images may also be chosen for highlighting on the journal table of contents or JCB homepage carousel. Images should be uploaded as TIFF or EPS files and must be at least 300 dpi resolution.

It is JCB policy that if requested, original data images must be made available to the editors. Failure to provide original images upon request will result in unavoidable delays in publication. Please ensure that you have access to all original data images prior to final submission.

The license to publish form must be signed before your manuscript can be sent to production. A link to the license to publish form will be sent to the corresponding author only. Please take a moment to check your funder requirements before choosing the appropriate license.

Additionally, JCB encourages authors to submit a short video summary of their work. These videos are intended to convey the main messages of the study to a non-specialist, scientific audience. Think of them as an extended version of your abstract, or a short poster presentation. We encourage first authors to present the results to increase their visibility. The videos will be shared on social media to promote your work. For more detailed guidelines and tips on preparing your video, please visit https://rupress.org/jcb/pages/submission-guidelines#videoSummaries.

Thank you for your attention to these final processing requirements. Please revise and format the manuscript and upload materials within 14 days. If you need an extension for whatever reason, please let us know and we can work with you to determine a suitable revision period.

Please contact the journal office with any questions at cellbio@rockefeller.edu.

Thank you for this interesting contribution, we look forward to publishing your paper in Journal of Cell Biology.

Sincerely,

Arshad Desai
Monitoring Editor
Journal of Cell Biology

Gabriele Stephan
Scientific Editor
Journal of Cell Biology

Reviewer #1:

Authors have answered all my comments so I am fully satisfied with the revised version of the work. It is a really pleasant and nice work, well done!

Reviewer #2:

The authors have done an excellent job addressing all my comments. They have made changes to the manuscript and added some data. This is an excellent manuscript.
