# Supplementary material for: Slender lobes is a spindle matrix protein regulated by spindle envelope kinase Greatwall in oocytes
Source: J Cell Biol. 2026 Oct 26;226(1):e202606020. doi: 10.1083/jcb.202606020 (PMC13701656; doi:10.1083/jcb.202606020)

Source Data Figure 4

A

kDa
170
130
100
70
55
40

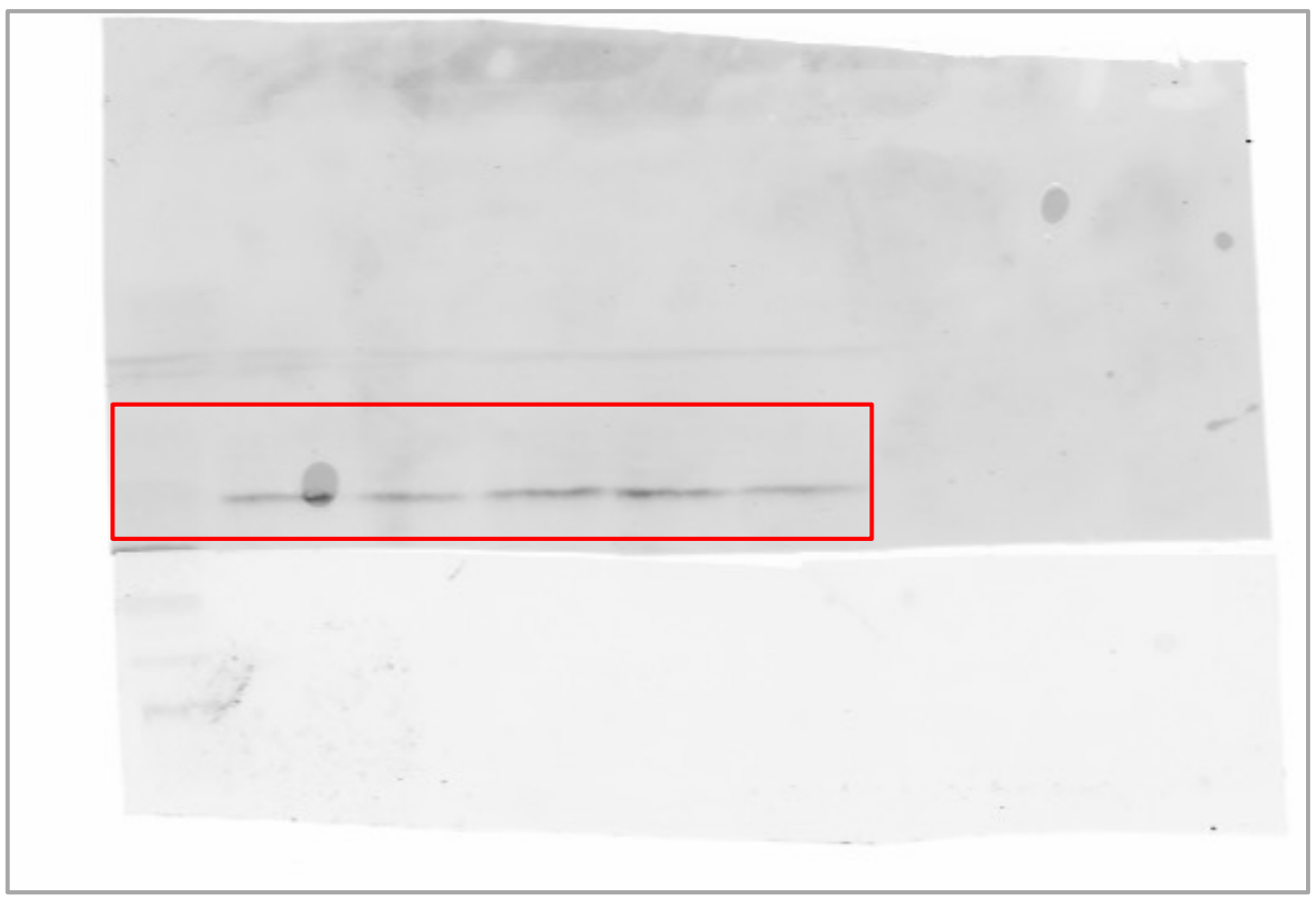

MW control B 1 2 3

sle RNAi

sle RNAi

MW control B 1 2 3

kDa
170
130
100
70
55

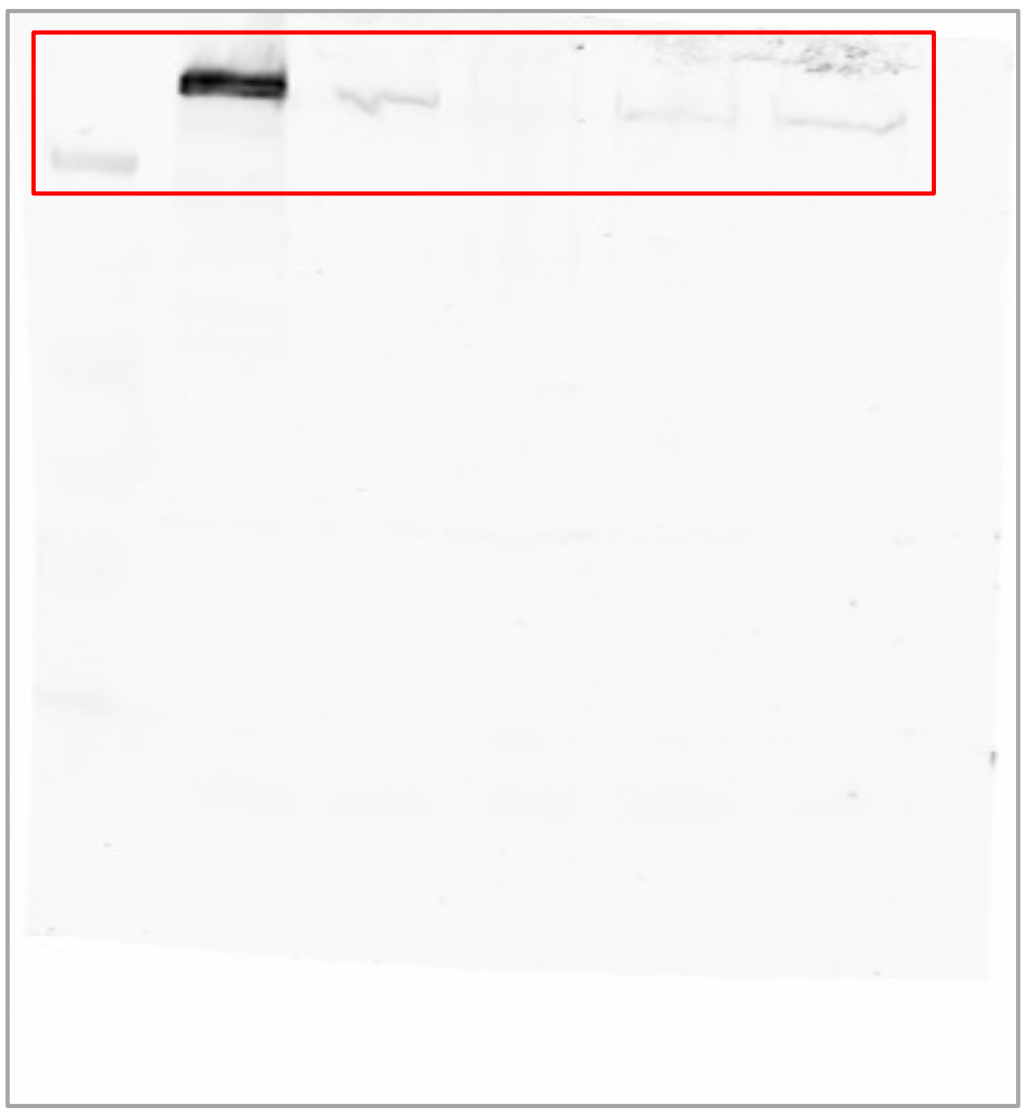

Supplement: Supplementary file 2 [file jcb_202606020_sourcedataf4.pdf]
