# Supplementary material for: Slender lobes is a spindle matrix protein regulated by spindle envelope kinase Greatwall in oocytes
Source: J Cell Biol. 2026 Oct 26;226(1):e202606020. doi: 10.1083/jcb.202606020 (PMC13701656; doi:10.1083/jcb.202606020)

Source Data Figure S3

A

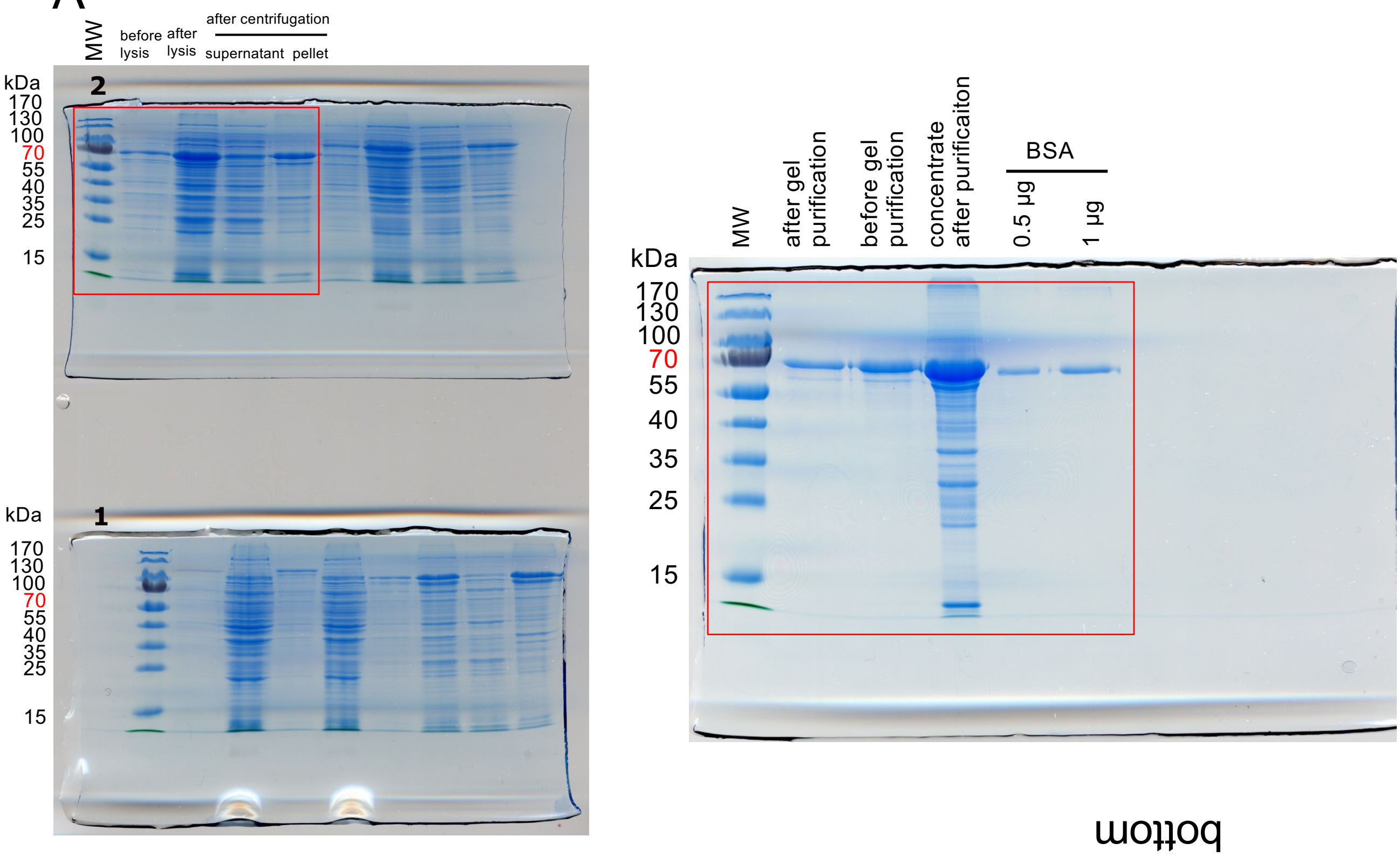

B

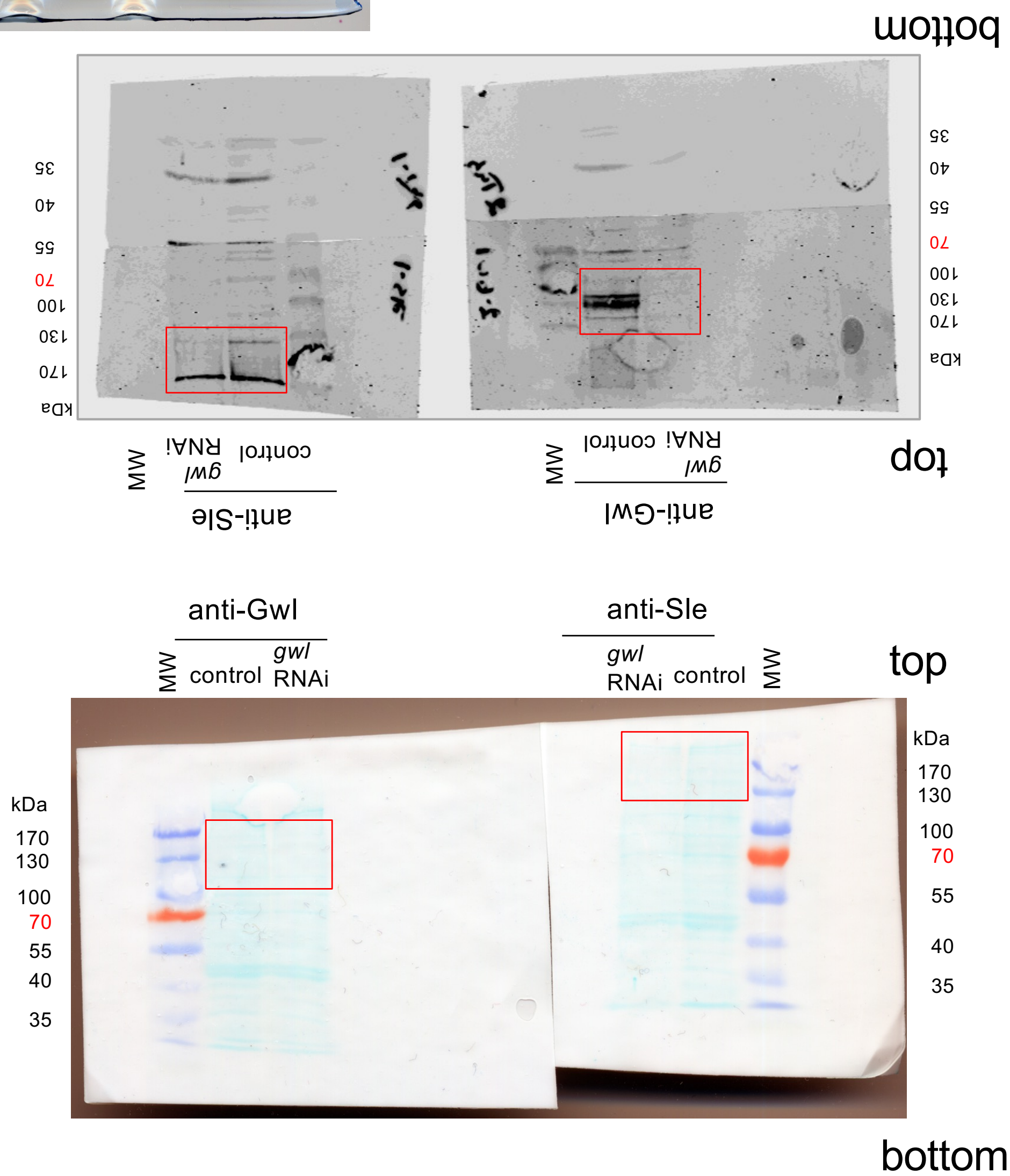

Supplement: Supplementary file 4 [file jcb_202606020_sourcedatafs3.pdf]
